# Neutrophils Promote Mycobacterial Trehalose Dimycolate-Induced Lung Inflammation via the Mincle Pathway

**DOI:** 10.1371/journal.ppat.1002614

**Published:** 2012-04-05

**Authors:** Wook-Bin Lee, Ji-Seon Kang, Ji-Jing Yan, Myeong Sup Lee, Bo-Young Jeon, Sang-Nae Cho, Young-Joon Kim

**Affiliations:** 1 Department of Biochemistry, College of Life Science and Biotechnology, Yonsei University, Seoul, Republic of Korea; 2 Department of Microbiology and Institute of Immunology and Immunological Disease, Yonsei University College of Medicine, Seoul, Republic of Korea; 3 Department of Integrated Omics for Biomedical Science, WCU Program of Graduate School, Yonsei University, Seoul, Republic of Korea; University of New Mexico, United States of America

## Abstract

Trehalose 6,6′-dimycolate (TDM), a cord factor of *Mycobacterium tuberculosis* (Mtb), is an important regulator of immune responses during Mtb infections. Macrophages recognize TDM through the Mincle receptor and initiate TDM-induced inflammatory responses, leading to lung granuloma formation. Although various immune cells are recruited to lung granulomas, the roles of other immune cells, especially during the initial process of TDM-induced inflammation, are not clear. In this study, Mincle signaling on neutrophils played an important role in TDM-induced lung inflammation by promoting adhesion and innate immune responses. Neutrophils were recruited during the early stage of lung inflammation following TDM-induced granuloma formation. Mincle expression on neutrophils was required for infiltration of TDM-challenged sites in a granuloma model induced by TDM-coated-beads. TDM-induced Mincle signaling on neutrophils increased cell adherence by enhancing F-actin polymerization and CD11b/CD18 surface expression. The TDM-induced effects were dependent on Src, Syk, and MAPK/ERK kinases (MEK). Moreover, coactivation of the Mincle and TLR2 pathways by TDM and Pam3CSK4 treatment synergistically induced CD11b/CD18 surface expression, reactive oxygen species, and TNFα production by neutrophils. These synergistically-enhanced immune responses correlated with the degree of Mincle expression on neutrophil surfaces. The physiological relevance of the Mincle-mediated anti-TDM immune response was confirmed by defective immune responses in Mincle^−/−^ mice upon aerosol infections with Mtb. Mincle-mutant mice had higher inflammation levels and mycobacterial loads than WT mice. Neutrophil depletion with anti-Ly6G antibody caused a reduction in IL-6 and monocyte chemotactic protein-1 expression upon TDM treatment, and reduced levels of immune cell recruitment during the initial stage of infection. These findings suggest a new role of Mincle signaling on neutrophils during anti-mycobacterial responses.

## Author Summary

Tuberculosis is one of the world's most pernicious diseases. *Mycobacterium tuberculosis* (Mtb), the causative agent of tuberculosis, has a lipid-rich cell wall that contains immunostimulatory properties. One of the lipid cell wall components, trehalose 6,6′-dimycolate (TDM), is a Mincle ligand and an immunogenic factor of Mtb that induces inflammatory responses leading to granuloma formation. Defining the major target and cellular functions of TDM may be requisite for delaying or preventing mycobacterial TDM-induced inflammation. Here, we demonstrated that neutrophils are important for the early phase of TDM-induced lung inflammation. Neutrophils are recruited during the initial stage of TDM-induced lung inflammation and Mincle is required for neutrophil access to TDM-challenged sites by enhancing neutrophil integrin expression, cytoskeleton remodeling, and cell adhesion. Furthermore, neutrophils aggravate TDM-induced lung inflammation by producing proinflammatory cytokines/chemokines. These findings open new perspectives for the role of Mincle signaling on neutrophils during TDM-induced inflammatory responses.

## Introduction

*Mycobacterium tuberculosis* (Mtb) is estimated to infect one-third of the world's population and is one of the most common causes of death by infectious diseases [1]. Infection by this bacterium mainly results in pulmonary disease, specifically the formation of granulomas, which are intended to wall-off the resistant bacteria. Initially, the granulomas consist of a center of infected macrophages surrounded by a mass of recruited monocytes and neutrophils. After lymphocytes arrive and acquired immunity develops, the granulomas attain delineated peripheral structures [2], [3]. Although the exact mechanisms of granuloma development underlying early immune responses have not been fully elucidated, it is believed that the local interaction of bacteria and host immune cells promotes local inflammation toward granuloma formation.

Diverse bacterial pathogen-associated molecular patterns (PAMPs) are thought to be involved in Mtb pathogenesis. Of the Mtb glycolipid cell wall components, trehalose 6,6′-dimycolate (TDM) is the most abundant lipid produced by virulent Mtb. TDM possesses immunostimulatory properties, including granulomagenesis and adjuvant activity for cell-mediated and humoral immune responses [1], [4]. In mice, purified TDM causes immunopathologies, including the release of proinflammatory cytokines and the formation of granulomas similar to those observed during Mtb infections [5]. Thus, how TDM induces inflammatory responses upon Mtb infection is a key question that must be addressed.

Cells of the innate immune system detect PAMPs through germline-encoded pattern recognition receptors (PRRs) [6]. Currently, four different classes of PRRs have been identified: (1) Toll-like receptors (TLRs), (2) RIG-I like receptors, (3) Nod-like receptors, and (4) C-type lectin receptors (CLRs). Among the PRRs, CLRs compose the largest family of cell-surface molecules with a carbohydrate-recognition domain [7]. Recently, Mincle (Clec4e, Clecsf9), belonging to the CLR family, was found to recognize TDM, as well as a synthetic derivative, trehalose 6,6-dibehenate [8], [9]. Mincle also recognizes various pathogens, such as *C. albicans*, *Malasezzia spp.*, *F. pedrosoi*, and an endogenous ligand, SAP130, from dead cells [10]–[13]. In macrophages, activated Mincle selectively associates with the immunoreceptor tyrosine-based activation motif-containing Fc receptor common γ-chain (FcRγ). The Mincle-FcRγ complex activates Syk kinase through an immunoreceptor tyrosine-based activation motif. This signaling event leads to heterotypic aggregation of Card9 with the adaptor protein Bcl10 and paracaspase Malt1, triggering the production of TNFα, IL-6, and macrophage inflammatory protein (MIP)-2 [13], [14]. Therefore, TDM-activated macrophages can induce cytokine/chemokine production, which can trigger robust recruitment and activation of inflammatory effector cells leading to pulmonary granuloma formation [15]. Thus, the activation of the Mincle signaling pathway in macrophages may be a key event in granuloma formation.

However, during mycobacterial infections, neutrophils and other immune cells are also augmented in the infected lung. Neutrophils become the predominant cell type infected by rapidly-replicating intracellular Mtb in patients with tuberculosis, which results in the over-activation of IFNγ and type-I IFN [16], [17]. Human neutrophils stimulated by Mtb produce several proinflammatory cytokines and chemokines, such as TNFα, IL-1β, IL-8, and MIP-1α [18], [19]. These results indicate that neutrophils, in addition to macrophages, can initiate a key effector response to Mtb. However, the mechanisms by which neutrophils regulate the initial stage of mycobacterial infections are not fully understood.

To understand the inflammatory responses of the initial phase of mycobacterial infections, we explored the role of Mincle signaling on neutrophils during TDM-induced lung inflammation and the subsequent immune responses. We demonstrated that neutrophils were recruited during the initial phase of TDM-induced inflammation in a Mincle-dependent manner. TDM-induced Mincle signaling on neutrophils resulted in surface expression of the CD11b/CD18 integrin, thereby augmenting neutrophil adhesion. TDM-induced Mincle signaling was dependent on Src, Syk, MAPK/ERK kinases (MEK), and MAPK. Moreover, coactivation of the Mincle and TLR2 pathways caused neutrophils to be in a highly-activated state through the induction of robust inflammatory responses, including high CD11b/CD18 surface expression, adhesion, ROS production, and TNFα production. The physiological relevance of the TDM-induced immune response was confirmed by the requirement of Mincle for efficient eradication of Mtb upon aerosol infection and by the defects of the neutrophil-depleted mice in the production of key cytokines/chemokines during TDM-induced inflammation. These results indicate that the Mincle pathway in neutrophils plays an important role in mycobacterial TDM-induced lung inflammation.

## Results

### Neutrophils were actively recruited to lung tissue during the initial stage of a TDM-induced granuloma model

**Figure 1:**
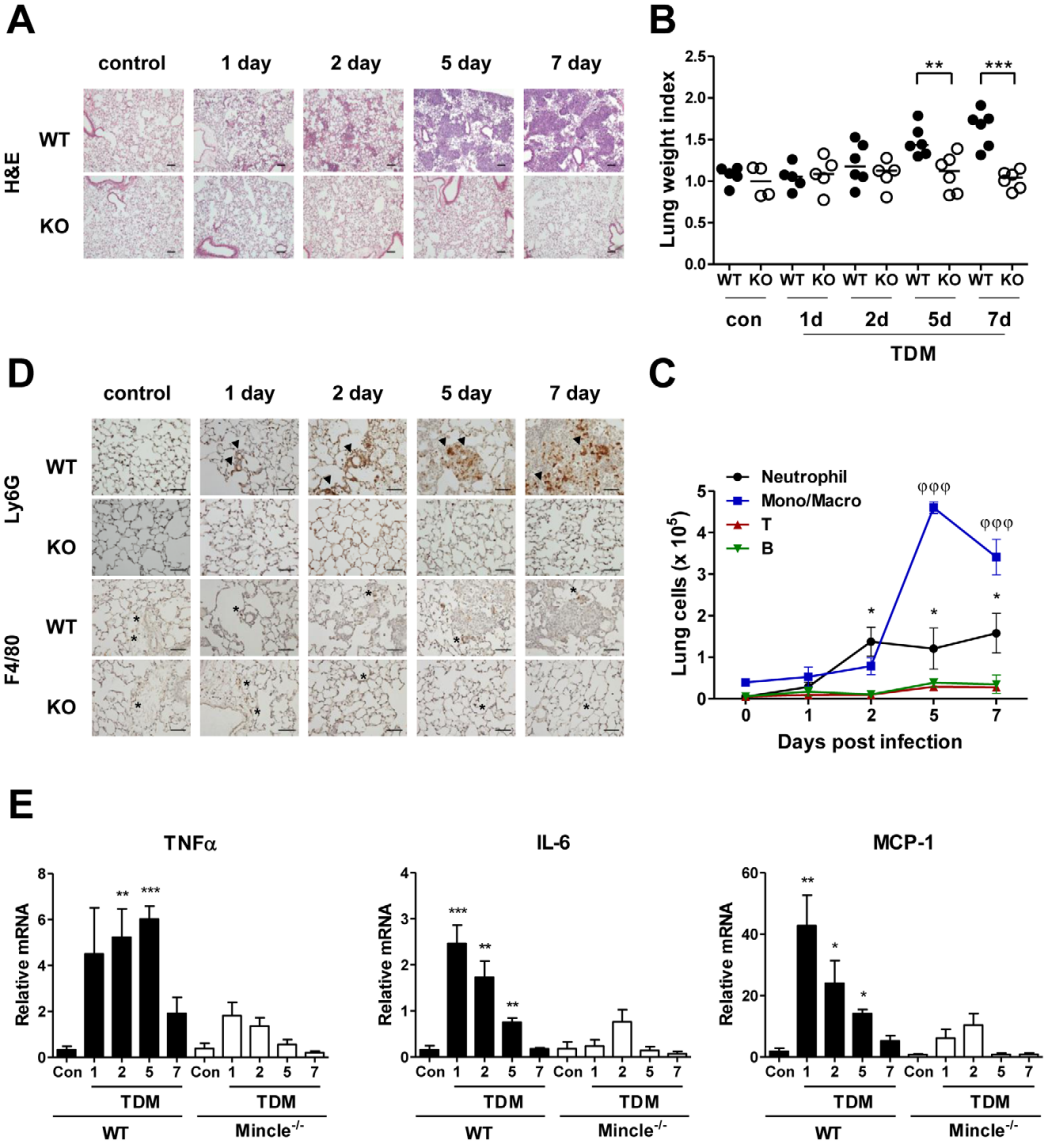
Kinetics of TDM-induced lung granuloma formation in WT and Mincle^−/−^ mice. Wild-type (WT) and Mincle^−/−^ mice were injected intravenously with an oil-in-water emulsion containing TDM. Emulsion without TDM was injected as a vehicle control. Mice were sacrificed at days 0, 1, 2, 5, and 7 post-TDM challenge. (A) Hematoxylin and eosin (H&E)-stained lung histology. Original magnification was 10×. Scale bars represent 100 µm. (B) Lungs from TDM-challenged mice were removed each day and inflammatory intensities were measured by calculating the lung weight index (LWI). n = 4–6 mice per group. Statistical significance: **p<0.01 and ***p<0.001. (C) Identification of leukocyte subsets in lung granulomas by flow cytometry. The number of neutrophils (CD11b^+^ Ly6G^+^), monocytes and macrophages (Mono/Macro, CD11b^+^ Ly6G^−^), T cells (CD3^+^), and B cells (CD19^+^) are indicated. Statistical significance is shown relative to day 0 for each group. *p<0.05 (in neutrophils) and ΦΦΦp<0.001 (in monocytes and macrophages). (D) Immunohistochemical Ly6G staining of neutrophils. Arrow heads indicate Ly6G+ cells near blood vessels (upper panels). Immunohistochemical F4/80 staining of macrophages. Asterisks indicate individual F4/80+ cells (lower panels). Sections are representative of 4–6 mice per group. Original magnification was 40×. Scale bars represent 50 µm. (E) TNFα, IL-6, and MCP-1 mRNA levels in whole-lung cell homogenates from WT and Mincle^−/−^ mice following TDM administration were measured by quantitative RT-PCR. Statistical significance is shown relative to control (con) for each group. *p<0.05, **p<0.01 and ***p<0.001. Data represent means ± SEM from five independent experiments.

A single dose of TDM in mice results in the development of lung granulomas that peak in number and size after 7 days and slowly resolve afterward [20]. Thus, this model provides an opportunity to investigate the recruitment of immune cells during the initial phase of inflammation leading to granuloma development. To this end, WT and Mincle^−/−^ mice were intravenously injected with a TDM water-in-oil emulsion. Lung tissues were analyzed 1, 2, 5, and 7 days after the challenge. WT mice formed transient granulomas after TDM injection (Figure 1A). Small focal clusters were noticeable 2 days post-TDM administration. The clusters became more complex by days 5 and 7, increasing in both size and number. However, Mincle^−/−^ lung tissue showed no detectable changes following the TDM injection. TDM-induced inflammatory lung swelling, as assessed by lung weight index, was augmented on days 5 and 7 post-TDM administration in WT mice, but was not discernible in Mincle^−/−^ mice (Figure 1B).

To enumerate the various leukocytes recruited to lungs of WT mice treated with a single TDM dose, flow cytometric analyses were performed with viable lung cells. The number of neutrophils (CD11b^+^, Ly6G^+^) was elevated on day 2 post-TDM administration, and was maintained through day 7 (Figure 1C). The numbers of monocytes and macrophages (CD11b^+^, Gr-1^+^, Ly6G^−^) were abruptly increased on day 5 after challenge. B-cell (CD19^+^) and T-cell (CD3^+^) numbers were not altered throughout the observed period. To confirm the abundance of recruited immune cells, lung sections were analyzed using immunohistochemistry with neutrophil (Ly6G^+^) and mature macrophage (F4/80^+^) markers (Figure 1D). Ly6G^+^ neutrophils accumulated around blood vessels on the day after TDM administration, and were maintained in WT mice. Mincle^−/−^ lungs did not contain recruited cells at any point. However, the number of F4/80^+^ cells, which represent the resident alveolar macrophages, remained constant during granuloma development in WT mice (Figure 1D, F4/80), even though a vast number of monocytes (CD11b^+^, Gr-1^+^, Ly6G^−^) infiltrated the lung on day 5 after TDM challenge (Figure 1C). Mincle^−/−^ lung tissues did not indicate increased levels of F4/80^+^ cells. These results suggest that two major types of effector cells, neutrophils and monocytes, are involved in the TDM-induced inflammation that leads to granuloma formation, and that neutrophils react immediately to TDM prior to monocytes.

To examine the physiological changes associated with immune cell recruitment, we measured the transcript levels of proinflammatory cytokines/chemokines during TDM-induced lung inflammation in WT and Mincle^−/−^ mice. In WT mice, IL-6 and MCP-1 expression peaked at day 1, and TNFα expression was elevated for a prolonged period (Figure 1E). Although the induction pattern of TNFα assimilated with the recruitment kinetics of monocytes, the early expression of IL-6 and MCP-1 correlated with the recruitment of neutrophils during the initial stage of inflammation. Taken together with the constant level of mature macrophages at infection sites, these results indicate that neutrophils could be the major source of the key inflammatory cytokines produced during the early stage of TDM-induced inflammation.

### Mincle expression on neutrophils was critical for their recruitment to TDM-challenged sites

**Figure 2:**
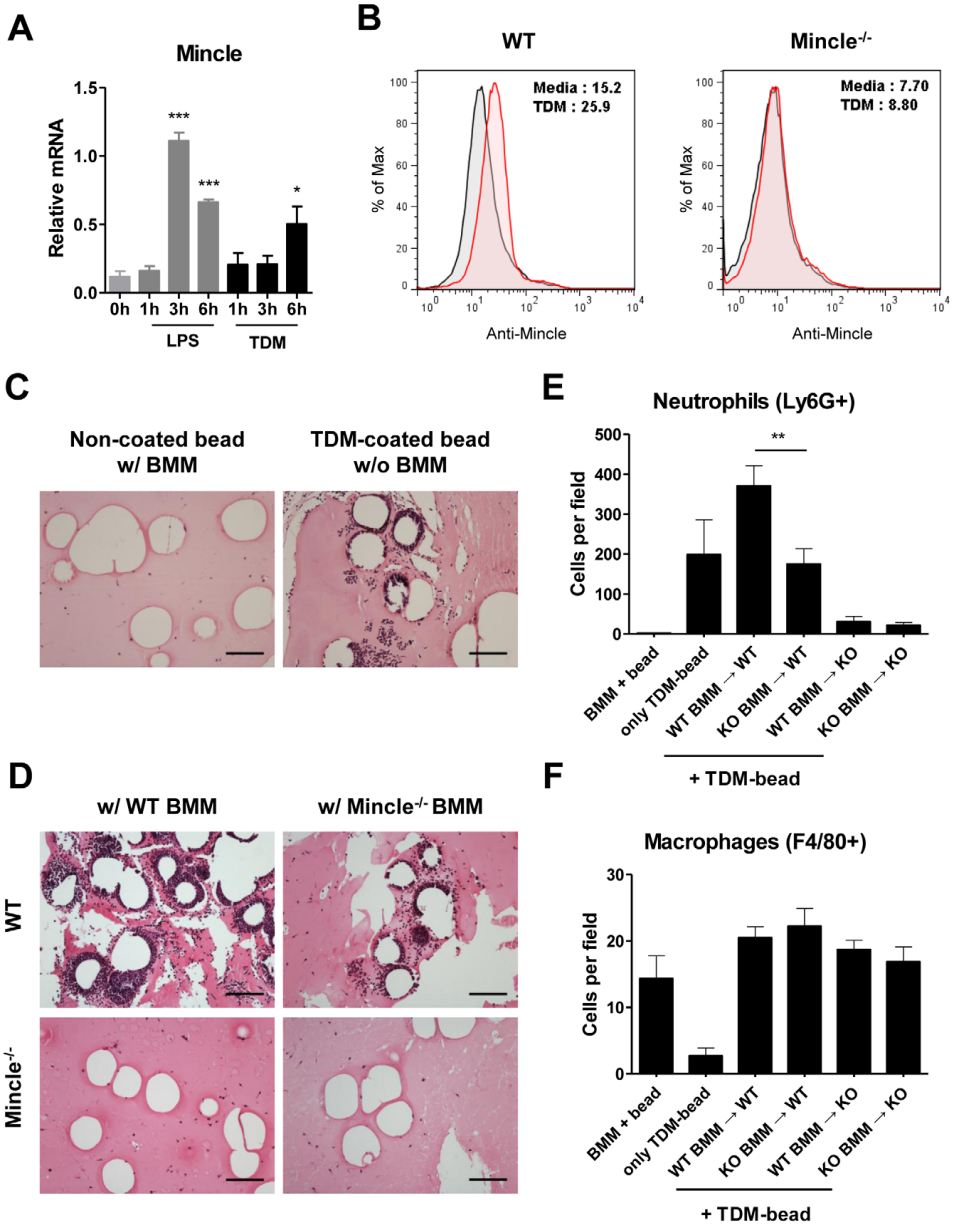
Neutrophils were recruited to TDM-coated-beads by recognition of TDM through Mincle. (A) Bone marrow (BM) neutrophils from C57BL/6 mice were stimulated with 10 ng/ml LPS or 25 µg/ml trehalose dimycolate (TDM). Mincle mRNA expression was measured by quantitative RT-PCR and normalized to Hprt mRNA levels. Significantly different levels from 0 h are indicated. *p<0.05 and ***p<0.001. (B) Mincle surface expression was determined by flow cytometry. BM neutrophils from WT and Mincle^−/−^ mice were stimulated with 25 µg/ml TDM for 18 h and analyzed by flow cytometry. See also Figure S1. (C–D) Matrices were injected into mice (s.c.) and harvested after 20 h. Formaldehyde-fixed paraffin-embedded sections were Hematoxylin and eosin stained. (C) Matrices contained non-coated beads with C57BL/6 bone marrow macrophages (BMMs) (left panel) or TDM-coated beads without BMMs (right panel). (D) Matrices containing TDM-coated beads were injected into wild-type (WT, upper panels) or Mincle^−/−^ (KO, lower panels) mice with WT BMMs (left panels) or Mincle^−/−^ BMMs (right panels). Photomicrographs are representative regions from each section. Scale bars represent 100 µm. (E–F) Ly6G^+^ neutrophils (E) or F4/80^+^ macrophages (F) adjacent to TDM-coated beads were counted from at least five randomly-selected fields from each stained section. Statistical significance: **p<0.01. Data are representative of at least three independent experiments.

Although several current models suggest that neutrophils are recruited to infection sites by chemokines released from activated macrophages, the concurrent recruitment of neutrophils with the immediate release of the key inflammatory cytokines suggests direct recruitment of neutrophils by TDM, possibly through recognition by Mincle. Although neutrophils express a vast repertoire of PRRs, including Dectin-1 [21] and CLEC-2 [22], the expression of Mincle, a known TDM receptor, has not been previously reported. When we examined Mincle expression in neutrophils, Mincle mRNA was induced rapidly by lipopolysaccharide (LPS), a TLR4 agonist (Figure 2A). Intriguingly, TDM treatment also induced Mincle expression at the transcription and cell surface expression levels (Figure 2A–B and Figure S1). Thus, we hypothesized that neutrophils can recognize TDM directly through autonomous induction of Mincle.

To determine if neutrophils directly recognize TDM, we took advantage of the TDM-coated bead-induced granuloma model [2], [23], with a minor modification of adding activated bone-marrow macrophages (BMMs) to the beads to mimic the function of resident macrophages. Subcutaneous insertion of TDM-coated beads induced a strong neutrophil recruitment around the inoculated beads within 20 h even in the absence of the additionally-supplied BMMs. However, beads containing only activated BMMs failed to recruit neutrophils under the same conditions (Figure 2C). These results indicate that TDM, not activated resident macrophages, is the major determinant of neutrophil recruitment to the beads.

To confirm the requirement of Mincle for neutrophil recruitment, we repeated the above experiments in Mincle^−/−^ mice in the presence and absence of WT BMMs. Contrary to WT mice, Mincle^−/−^ mice inoculated with TDM-coated beads showed no neutrophil recruitment even in the presence of the supplemented WT BMMs. This indicates that Mincle expression by neutrophils is required for neutrophil recruitment to the beads. Although the WT BMMs were dispensable for neutrophil recruitment in the TDM-bead model, the BMMs appeared to enhance the recruitment of neutrophils to the beads in Mincle^−/−^ mice. In WT mice, beads mixed with WT BMMs induced higher levels of neutrophil recruitment than did beads mixed with Mincle^−/−^ BMMs (Figure 2D and 2E). Therefore, it is highly probable that BMMs enhanced the recruitment of neutrophils by releasing TDM-induced chemotactic factors in a Mincle-dependent manner. Under these conditions, the number of macrophages remained similar to that of the initially-inoculated BMMs, suggesting that macrophage recruitment was not affected by genetic backgrounds within 20 h (Figure 2F). Therefore, Mincle expression was necessary for neutrophils to infiltrate TDM-challenged sites, and the proinflammatory cytokines/chemokines produced by resident macrophages promoted their recruitment.

### TDM increased neutrophil adhesion and F-actin polymerization

**Figure 3:**
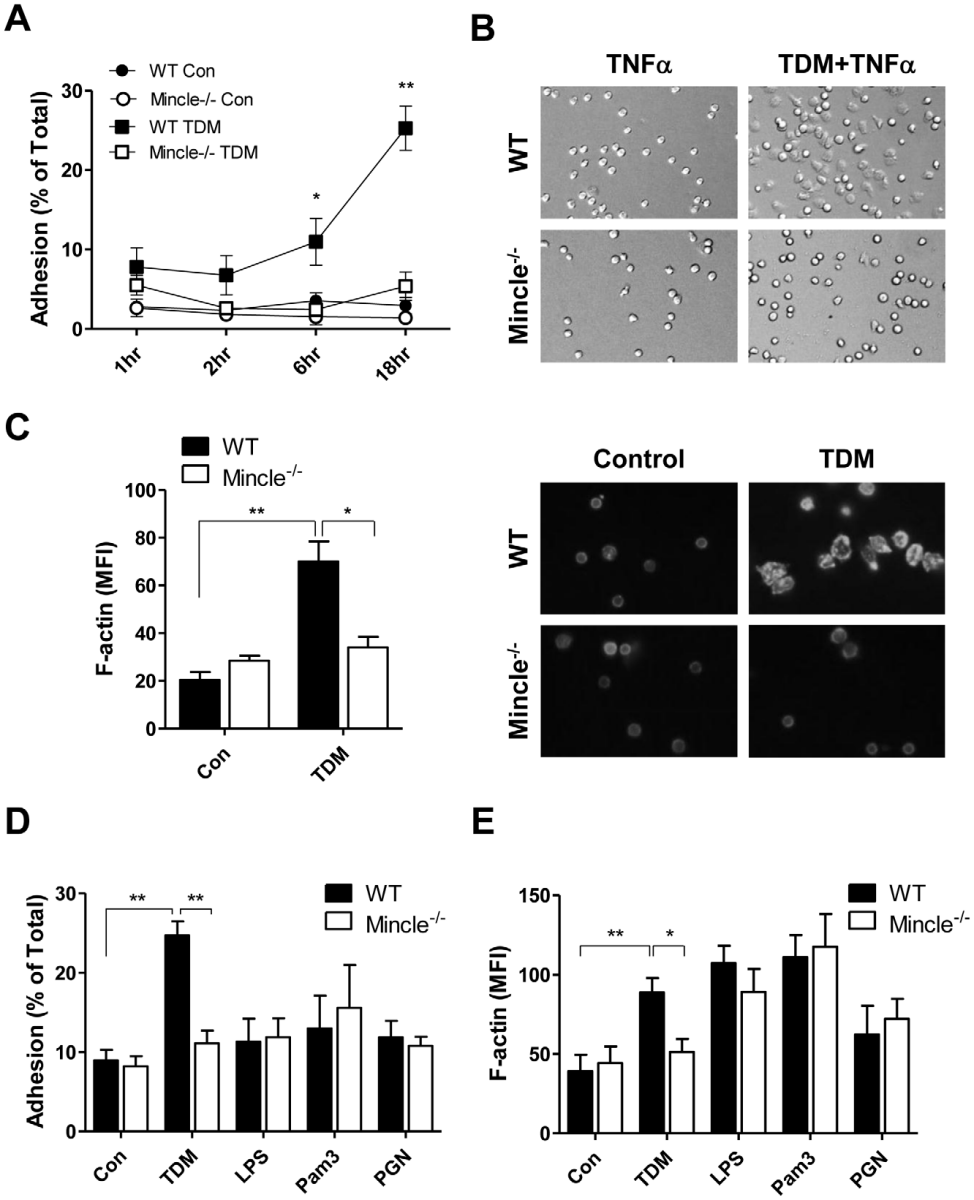
TDM-induced neutrophil adhesion and F-actin polymerization are Mincle dependent. (A) Firmly-adherent wild-type (WT) and Mincle^−/−^ bone marrow (BM) neutrophils were counted after incubation in the presence or absence of 25 µg/ml trehalose dimycolate (TDM). Statistical significance is shown relative to unstimulated control (con) for each group. *p<0.05 and **p<0.01. (B) Firmly-adherent WT and Mincle^−/−^ BM neutrophils treated with TNFα and/or TDM for 60 min were imaged by phage-contrast microscopy. Original magnification was 400×. (C) BM neutrophils were cultured on TDM-coated coverslips for 60 min, and F-actin polymerization was monitored by immunofluorescent microscopy (right). Images are representative of three independent experiments. Alexa 568-phalloidin staining was quantified by mean fluorescence intensity (MFI, left). Statistical significance: *p<0.05 and **p<0.01. (D–E) Neutrophil adherence (D) and F-actin polymerization (E) stimulated by TDM, LPS, Pam3CSK4, or peptidoglycan for 18 h (D) or 1 h (E). Statistical significance: *p<0.05 and **p<0.01. Data are expressed as means ± SEM from at least three independent experiments.

For the previous histology data (Figure 2C and 2D), neutrophils were recruited and adhered to the TDM-coated beads. Therefore, we examined whether neutrophil adhesion increased following TDM stimulation. WT neutrophils showed a gradual increase in adhesion (6 h), and reached a strong level of adhesion 18 h after TDM stimulation (Figure 3A and Figure S2). However, Mincle^−/−^ neutrophils showed no induction of adhesion upon TDM treatment. TNFα increases neutrophil activation [24], [25]. Therefore, we examined the results of TNFα/TDM co-stimulation of neutrophils. TNFα/TDM co-stimulated neutrophils adhered to plates and changed morphology more rapidly than did TNFα only stimulated neutrophils (Figure 3B).

To investigate whether TDM-induced adhesion accompanies actin remodeling, we stained TDM-stimulated neutrophils with fluorescently-labeled phalloidin and analyzed the cells by fluorescence microscope and flow cytometry. As a result, actin polymerization of WT neutrophils was increased by TDM stimulation, but which of Mincle^−/−^ neutrophils was not changed (Figure 3C and Figure S3). To test the specificity of Mincle on actin remodeling and cell adhesion, we examined the effect of Mincle mutations in response to other types of inflammatory stimuli, such as LPS, Pam3CSK4, and *E. coli* peptidoglycan. Although LPS and Pam3CSK4 treatments also induced strong actin remodeling in neutrophils, their effects on cell-adhesion activities were not comparable to those of TDM (Figure 3D and 3E). Additionally, Mincle was dispensable for the uncoupled cell-adhesion and actin remodeling induced by other PAMPs. These results support a specific requirement of Mincle signaling for neutrophil adhesion through F-actin polymerization.

### TDM-induced surface expression of CD11b/CD18 and enhanced neutrophil adhesion

**Figure 4:**
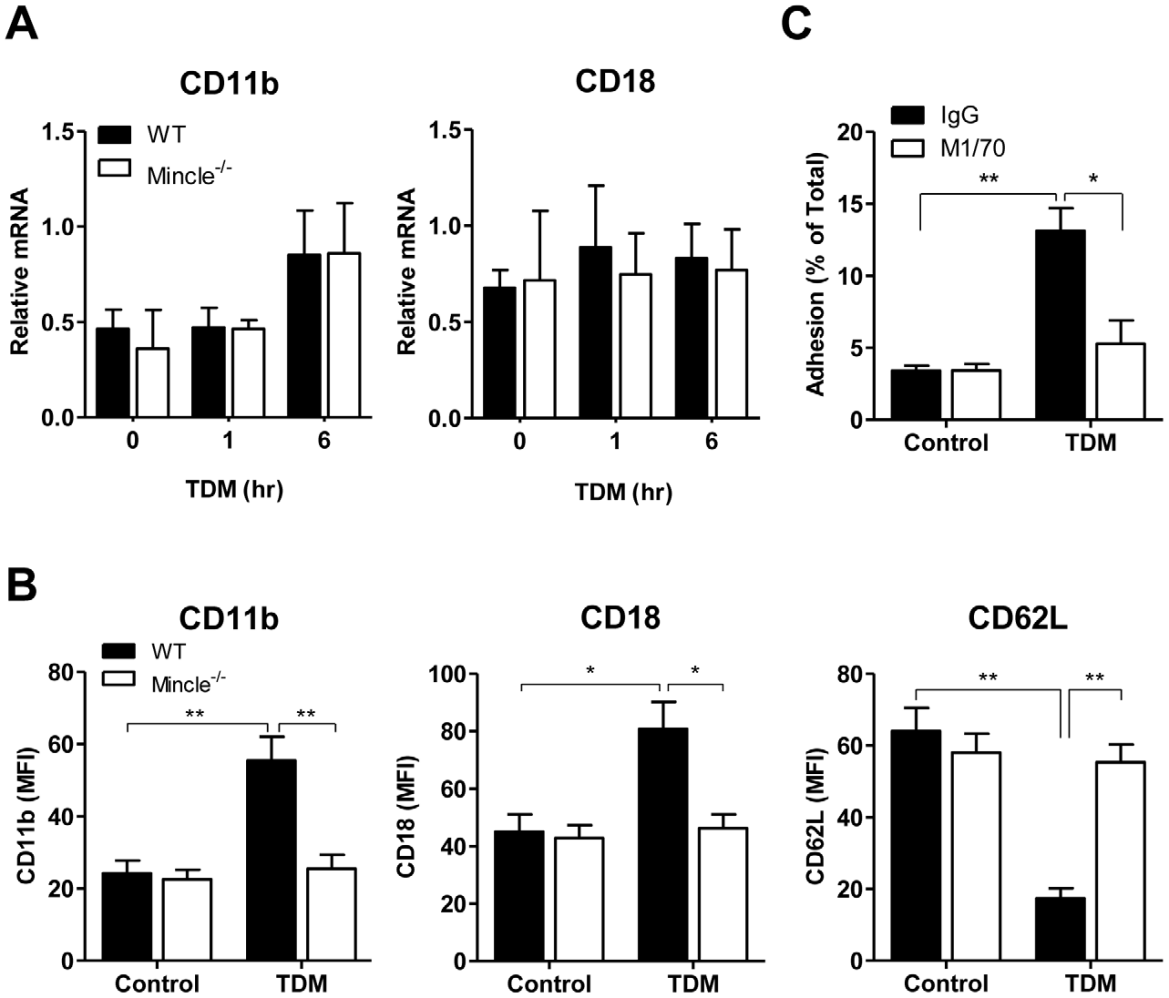
Up-regulated CD11b/CD18 surface expression following TDM-Mincle signaling enhanced neutrophil adhesion. (A) Bone marrow (BM) neutrophils from wild-type (WT) and Mincle^−/−^ mice were stimulated with 25 µg/ml trehalose dimycolate (TDM). CD11b and CD18 mRNA expression was measured by quantitative RT-PCR. (B) Surface CD11b, CD18, and CD62L levels of control or 18 h TDM stimulated BM neutrophils were quantified by flow cytometry. MFI, mean fluorescence intensity (MFI). Statistical significance: *p<0.05 and **p<0.01. (C) Firmly-adherent neutrophils were counted after incubation in the presence or absence of TDM and were treated with anti-CD11b (M1/70) or control immunoglobulin G (IgG). Statistical significance: *p<0.05 and **p<0.01. Data are expressed as means ± SEM from three independent experiments.

After neutrophil activation, cell spreading, cellular adhesion, and F-actin polymerization are mainly induced through CD11b/CD18 (α_M_β_2_-integrin, Mac-1) signaling [26], coincident with loss of CD62L (L-selectin) from the surface [27]. CD62L shedding regulates the velocity of leukocyte rolling in the early step of leukocyte-endothelial interaction [28]. Therefore, we examined induction of CD11b/CD18 and loss of CD62L following TDM treatment. The mRNA levels of CD11b and CD18 were not significantly affected by TDM treatment in WT and Mincle^−/−^ neutrophils (Figure 4A). However, TDM treatment induced a significant increase in CD11b/CD18 cell surface expression on WT neutrophils and the concurrent loss of CD62L (Figure 4B). Mincle^−/−^ neutrophils failed to up-regulate CD11b/CD18 and shed CD62L after TDM stimulation. To confirm CD11b/CD18-mediated neutrophil adhesion, we used a CD11b-specific function-blocking mAb (M1/70) in neutrophil adhesion experiments. Blocking CD11b/CD18 signaling resulted in impressive suppression of neutrophil adhesion in response to TDM (Figure 4C). These results indicate that TDM-induced neutrophil adhesion is mediated by the up-regulation of the inside-out CD11b/CD18 signaling.

### TDM-induced neutrophil adhesion required phosphorylation of Syk and MAP kinases

**Figure 5:**
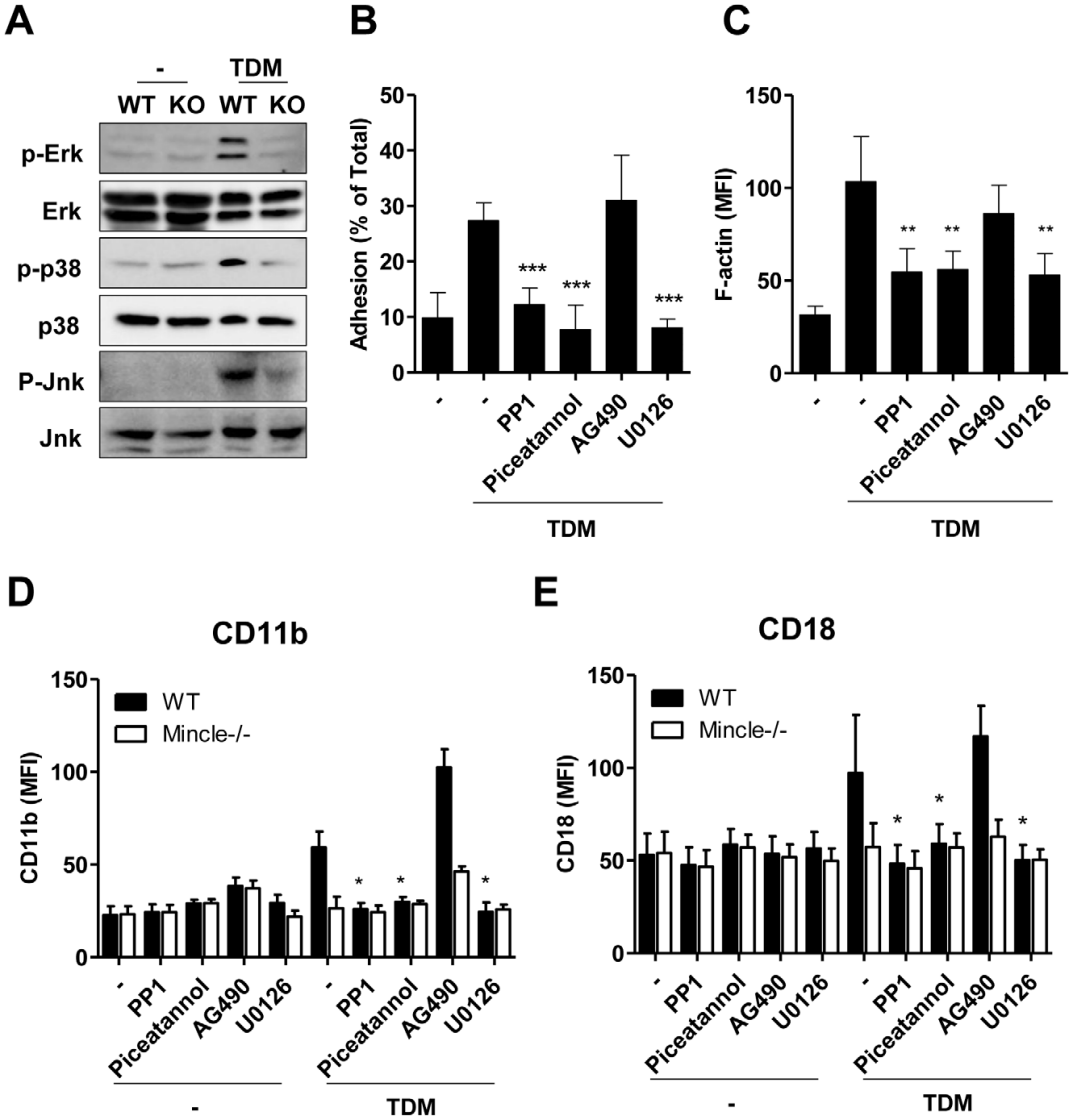
Activation of Syk and MAP kinase during TDM-mediated neutrophil adhesion. (A) Levels of phosphorylated Erk, p38, and Jnk in wild-type (WT) and Mincle^−/−^ (KO) bone marrow (BM) neutrophils 30 min after TNFα (Con) and TNFα/trehalose dimycolate (TDM) stimulation as determined by immunoblot analysis. (B–C) Neutrophil adherence (B) and F-actin polymerization (C) were measured in C57BL/6 BM neutrophils stimulated with TDM in the presence of PP1 (5 µM), Piceatannol (40 µM), AG490 (25 µM), or U0126 (10 µM). Statistical significance is shown relative to TDM-treated neutrophils. **p<0.01, and ***p<0.001 (D–E) Surface expression of CD11b (B) and CD18 (C) was measured by flow cytometry in WT and Mincle^−/−^ BM neutrophils stimulated with TDM for 18 h in the presence of specific inhibitors. Statistical significance is shown relative to TDM-treated WT neutrophils. *p<0.05. Data are expressed as means ± SEM from three independent experiments.

Previous studies show that actin remodeling associated with cell adhesion is regulated by MAP kinases activated by diverse receptors [29], [30]. However, Mincle signaling in macrophages has been suggested to involve the Src and Syk kinase pathways to produce TNFα and MIP-2 [13]. Therefore, we investigated whether similar pathways are used in TDM-mediated Mincle signaling during neutrophil adhesion. After TDM stimulation, WT neutrophils showed increased levels of tyrosine phosphorylation of Erk, p38, and Jnk MAP kinases, whereas Mincle^−/−^ neutrophils did not show altered tyrosine phosphorylation (Figure 5A). To confirm the requirement of these kinases for neutrophil adhesion, we used various kinase inhibitors to block cell adhesion. Pre-treating neutrophils with the Src kinase inhibitor PP1, the Syk kinase inhibitor Piceatannol, or the MAPK/ERK kinase (MEK) inhibitor U0126 blocked TDM-mediated cell adhesion (Figure 5B) and actin remodeling (Figure 5C) induced by TDM stimulation. Treatment with AG490, a Jak2 inhibitor, had no effect. The same kinase inhibitors also caused down regulation of CD11b/CD18 surface expression (Figure 5D and 5E). These findings suggest that activation of Src, Syk, and MAP kinases is essential for the up-regulation of CD11b/CD18 surface expression that leads to neutrophil adhesion following TDM stimulation.

### Co-stimulation of the TLR2 pathway greatly potentiated Mincle-mediated neutrophil responses

**Figure 6:**
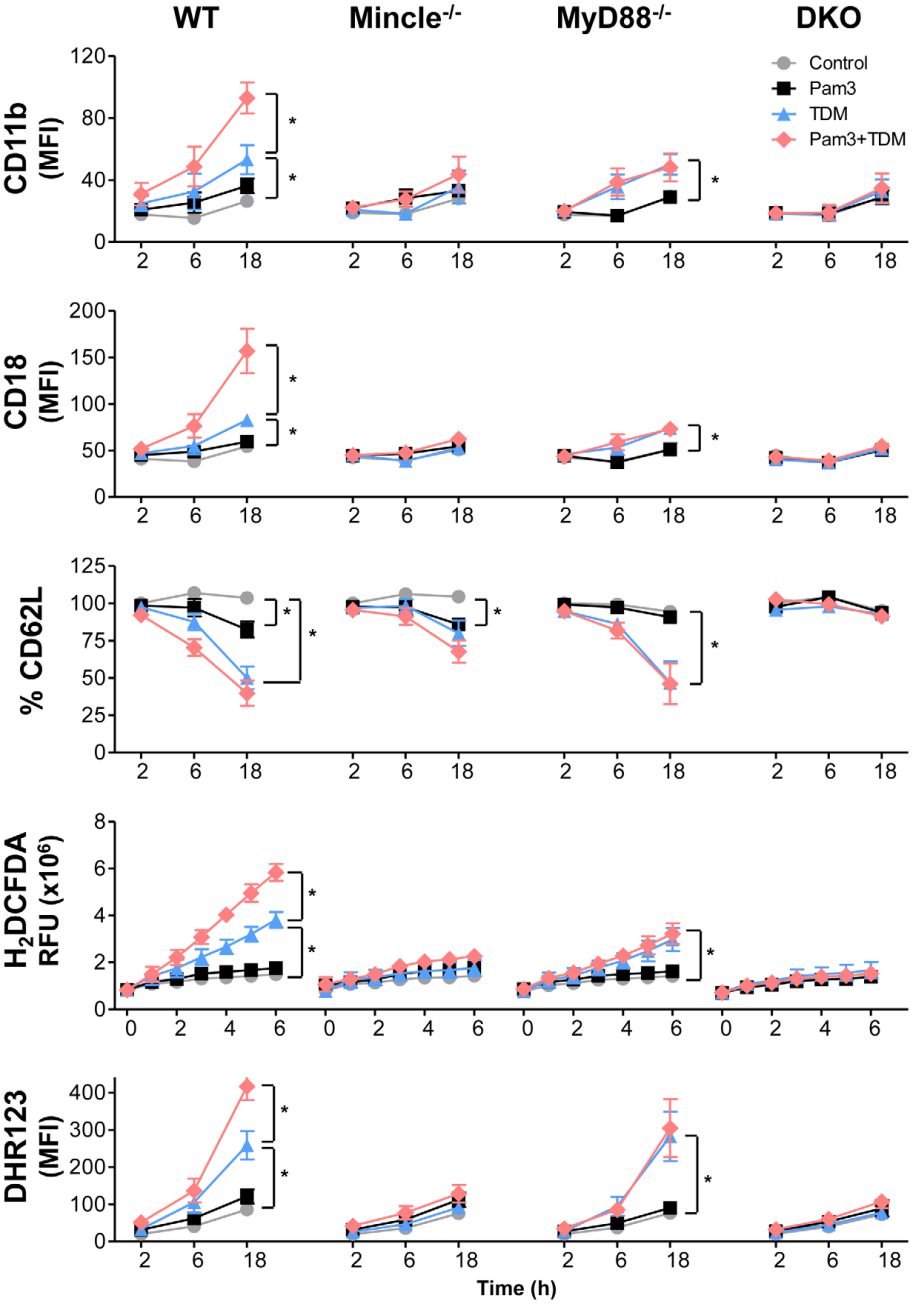
Co-stimulation with TDM and Pam3CSK4 synergistically up-regulated CD11b/CD18 expression and ROS production. Wild-type (WT), Mincle^−/−^, MyD88^−/−^, and double knockout (DKO) bone marrow neutrophils were stimulated with Pam3CSK4 (10 ng/ml) and/or trehalose dimycolate (TDM, 25 µg/ml) for the indicated times (h). Surface expression of CD11b, CD18, and CD62L was analyzed by flow cytometry. Reactive oxygen species (ROS) production was assessed by the oxidation of H_2_DCFDA derivatives as measured with a microplate reader at 485/520 nm and DHR123 as measured by flow cytometry. Statistical significance: *p<0.05. Data are expressed as means ± SEM from at least three independent experiments.

TLR2 is involved in recognition of Mtb, and TLR2 signaling through MyD88 plays an important role in the initiation of innate host defenses [31]. TLR2^−/−^ mice show defective granuloma formation, and are more susceptible to Mtb infection as compared to WT mice [32], [33]. Thus, coactivation of the TLR2 and Mincle pathways by distinct Mtb PAMPs is likely required for the full activation of neutrophil responses to Mtb infection. To dissect the contribution of these pathways to the response of activated neutrophils during Mtb infection, we analyzed the kinetic profiles of ROS production and surface expression of CD11b, CD18, and CD62L on neutrophils stimulated with Pam3CSK4 and/or TDM under various genetic background combinations of MyD88 and Mincle mutations (Figure 6). The up-regulation of released and intracellular ROS production and the up- and down-regulation of CD11b/CD18 and CD62L surface expression, respectively, by TDM-stimulated neutrophils were greatly enhanced following co-stimulation with Pam3CSK4. However, Pam3CSK4 treatment alone caused only a minor stimulatory effect on the neutrophils. Similar experiments in MyD88 or Mincle^−/−^ mice further confirmed that Mincle activation by TDM is the primary signaling pathway for neutrophil activation. MyD88^−/−^ neutrophils showed TDM-induced responses similar to those of WT neutrophils. Therefore, these responses likely occurred without the co-stimulatory effect of the activated TLR pathway.

**Figure 7:**
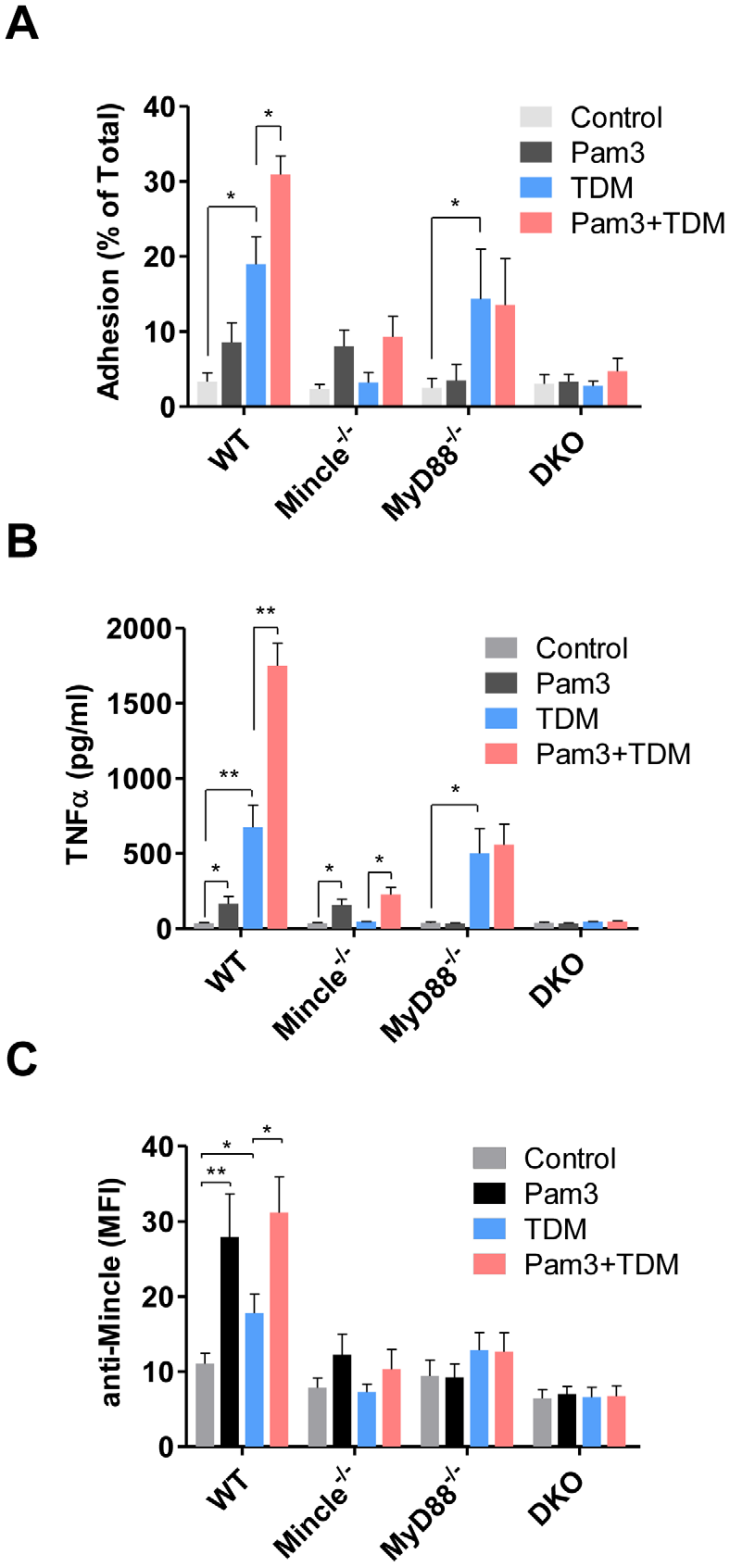
Cell adhesion and TNFα production following co-stimulation with TDM/Pam3CSK4 correlated with Mincle expression. Wild-type (WT), Mincle^−/−^, MyD88^−/−^, and Mincle^−/−^MyD88^−/−^ (DKO) bone marrow neutrophils were stimulated with Pam3CSK4 (10 ng/ml) and/or trehalose dimycolate (TDM, 25 µg/ml). (A) Firmly-adherent neutrophils were counted after incubation for 6 h with Pam3CSK4 and/or TDM. (B) TNFα production was measured 24 h after stimulation. (C) Mincle expression on neutrophils 18 h after stimulation was measured by flow cytometry. Statistical significance: *p<0.05 and **p<0.01. Data are expressed as means ± SEM from more than three independent experiments.

Moreover, WT and MyD88^−/−^ neutrophils induced cell adhesion and TNFα production by TDM stimulation, and WT and Mincle^−/−^ neutrophils produced TNFα by Pam3CSK4 (Figure 7A–B and Figure S4). However, co-stimulatory effects of cell adhesion and TNFα production were detected in only WT neutrophils. Thus, the primary requirement of the TDM-activated Mincle pathway in neutrophils was further confirmed by cell adhesion and TNFα production assays.

Mincle mRNA expression was induced by both LPS and TDM; however, the LPS treatment produced a much stronger and faster change in Mincle expression than did TDM stimulation (Figure 2A). This finding prompted us to examine whether the higher Mincle surface expression induced by TLR signaling caused the synergistic TDM-induced inflammatory responses on neutrophils. Indeed, Pam3CSK4 caused a strong induction of Mincle protein on neutrophils in a MyD88-dependent fashion (Figure 7C). Although TDM treatment induced Mincle expression on neutrophil surfaces, Pam3CSK4-mediated induction was the most rapid and primary source of Mincle overexpression. Therefore, TLR signaling likely potentiates the Mincle pathway by inducing Mincle surface expression, and the higher level of activation of the TDM-induced Mincle pathway promotes neutrophil adherence and ROS production.

### Mincle signaling was required for immune responses against Mtb infection

**Figure 8:**
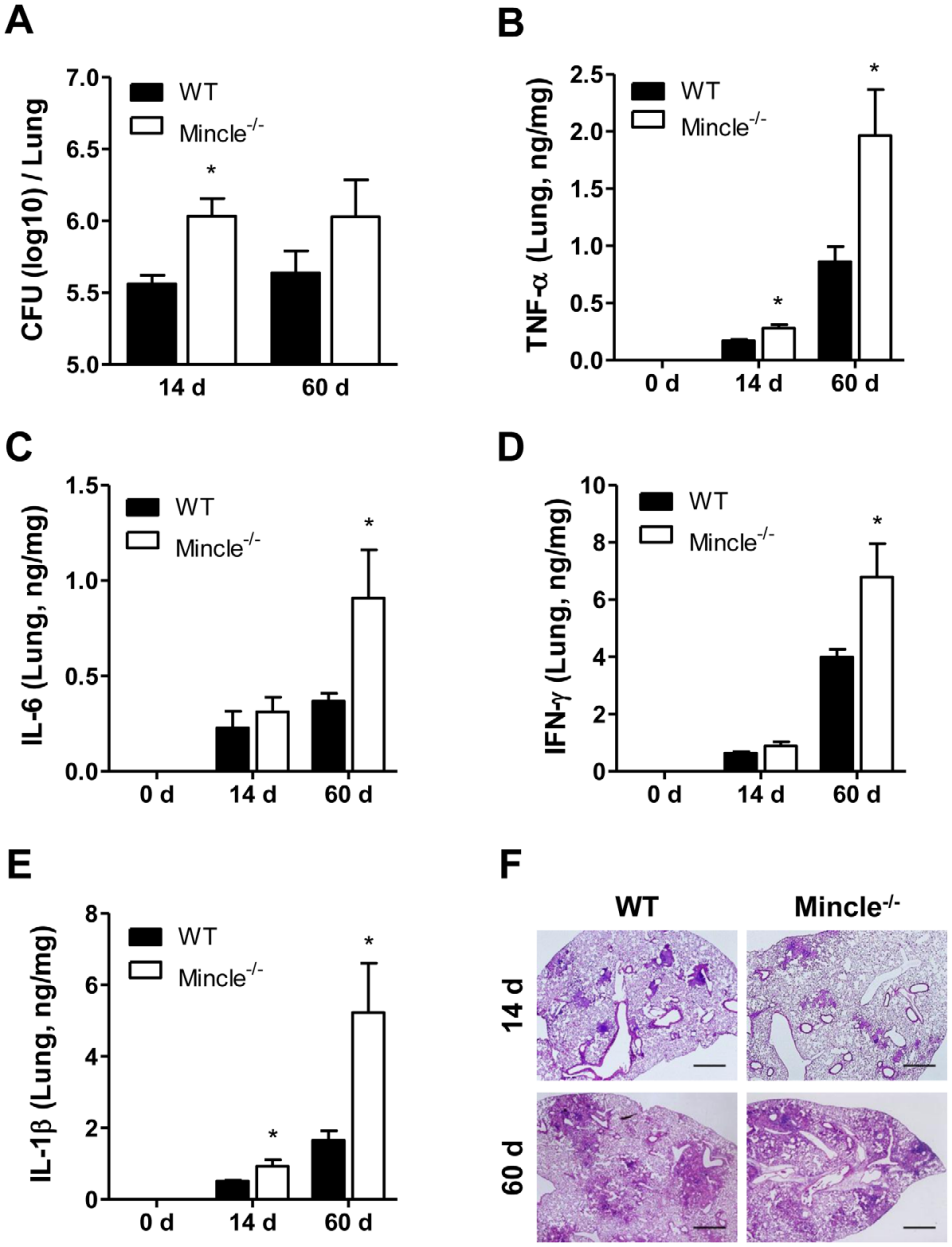
Bacterial loads and cytokine levels are elevated in lungs from Mincle^−/−^ mice following *Mycobacterium tuberculosis* infection. (A) Viable bacterial numbers in the lungs of wild-type (WT) and Mincle^−/−^ mice (n≥4) were determined at the indicated time points following *mycobacterium tuberculosis* (Mtb) infection. Mean log colony-forming units (CFUs) per lung (±SEM) are shown. (B–E) TNFα (B), IL-6 (C), IFNγ (D), and IL-1β (E) levels were measured in the lungs of WT and Mincle^−/−^ mice at the indicated time points following virulent Mtb strain Erdman infection. Statistical significance is shown relative to WT for each group. *p<0.05. (F) Histology of Hematoxylin and eosin (H&E)-stained lung tissues from Mtb-infected WT and Mincle^−/−^ mice. Original magnification was 4×. Scale bars represent 500 µm.

To validate the physiological significance of the *in vitro* results showing the essential role of Mincle in TDM-induced inflammation, we examined the requirement of Mincle for defense against mycobacterial infection in mice. WT and Mincle^−/−^ mice were aerosol-infected with approximately 100 CFU of Mtb. The Mtb load and the expression of key inflammatory cytokines in lung tissue were measured 2 and 8 weeks after the infection to examine the effect during innate and adaptive immune responses, respectively. As shown in Figure 8A, Mincle^−/−^ mice had a higher bacterial burden than did WT control mice at both time points, indicating a defect in the clearance of Mtb in Mincle^−/−^ mice. Although Mincle is required for the production of key inflammatory cytokines in an *in vitro* system, the mutant mice showed higher levels of proinflammatory cytokines, including TNFα, IL-6, IFNγ, and IL-1β, than did WT mice. These results likely reflect the indirect consequence of a higher bacterial burden in the Mincle mutant mice, which causes additional inflammatory responses, rather than reflecting the direct requirement of Mincle for the production of key inflammatory cytokines (Figure 8B–8E). Despite these differences, granuloma formation measured by H&E staining was similar in WT and mutant lungs infected with aerosolized Mtb (Figure 8F). Therefore, the higher inflammation levels in the mutants may be a result of the failure to clear the infected Mtb, rather than the development of granulomas. These data indicate that Mincle signaling is required to control Mtb proliferation.

### Neutrophils promoted lung inflammation *in vivo* following TDM-challenge in mice

**Figure 9:**
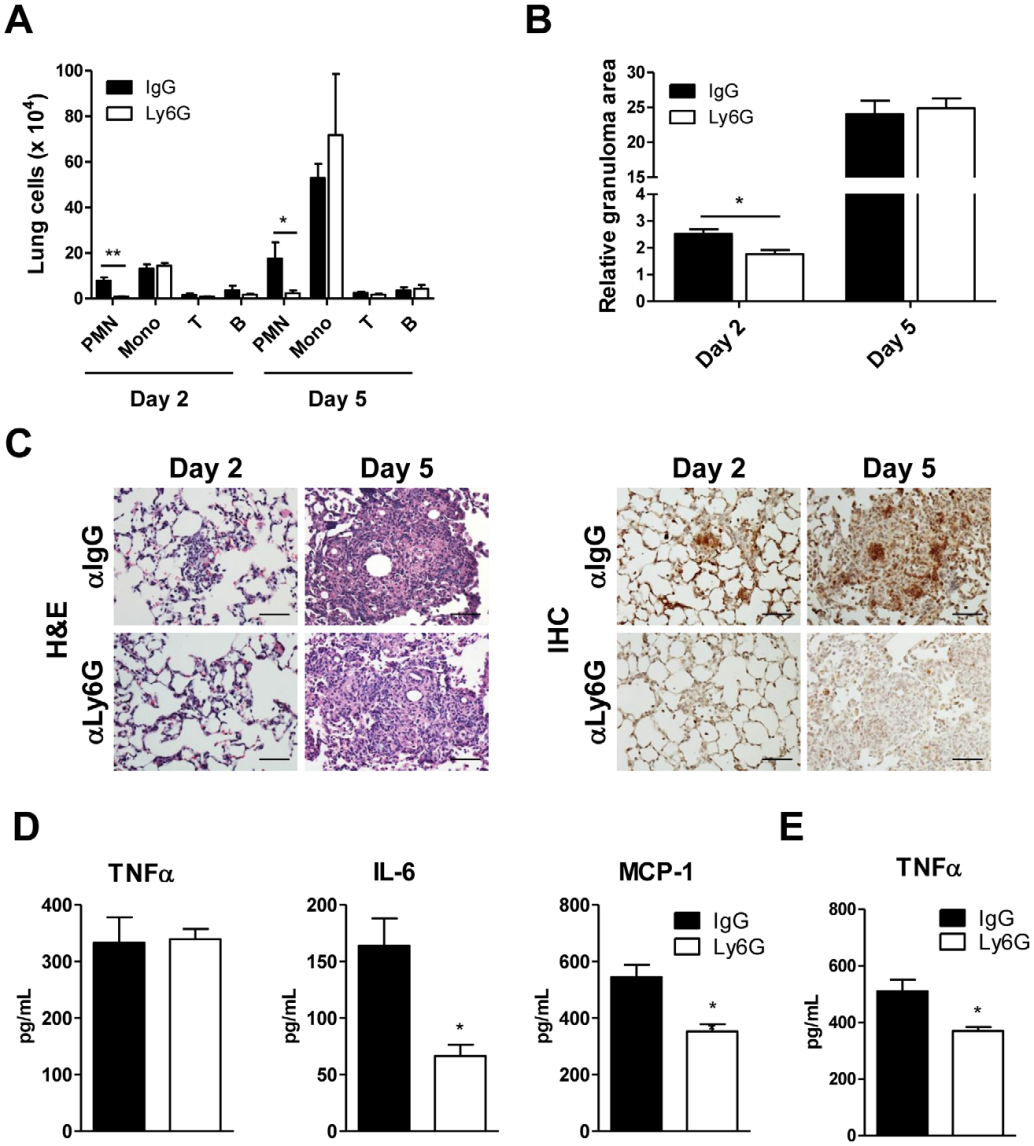
Neutrophil-depleted mice had weakened immunity during TDM-elicited inflammation. C57BL/6 mice received intravenous injections of Ly6G mAb or IgG control mAb 1 day before trehalose dimycolate (TDM) administration (n = 3 mice/group). After TDM administration (2 or 5 days), lung tissues and peripheral blood were obtained. (A) Whole-lung cells were analyzed using flow cytometry. Statistical significance: *p<0.05 and **p<0.01. (B) Image quantification of relative granuloma areas. Statistical significance: *p<0.05. (C) Hematoxylin and eosin (H&E) staining and Ly6G-immunohistochemical staining of neutrophils in lung tissues. Original magnification was 40×. Scale bars represent 50 µm. (D) TNFα, IL-6, and MCP-1 protein levels from whole-lung homogenates were determined by CBA. (E) Serum TNFα levels in peripheral blood from mice were measured by CBA. Statistical significance: *p<0.05. Data are expressed as means ± SEM from more than three independent experiments.

Once the physiological relevance of the Mincle-mediated anti-TDM response was established, we wanted to confirm the importance of Mincle signaling in neutrophils during TDM-driven lung inflammation. Our data hint at the importance of neutrophils in TDM-induced lung inflammation in that neutrophils accumulated in the infected sites before any other types of immune cells arrived (Figure 1), and that neutrophils induced strong immune responses against TDM (Figures 6 and 7). To investigate the effect on host immune responses by the early accumulation of neutrophils, we depleted neutrophils in mice using a Ly6G monoclonal antibody (1A8) that specifically-depletes neutrophils without impacting Gr-1^+^ monocyte populations [34]. Because antibody-mediated neutrophil-depleted mice regenerate their neutrophils within several days, this type of neutrophil depletion is not compatible with experiments investigating chronic Mtb-induced inflammation after 2 weeks to detect distinct immune responses. Thus, mice were intravenously injected with TDM during neutrophil depletion and the inflammatory responses in the lung were analyzed. Ly6G antibody injections were sufficient to maintain the neutrophil-depleted condition for at least five days. TDM administration led to rapid neutrophil recruitment into the lung tissue, followed by recruitment of a large number of monocytes in the control IgG-injected WT mice. On the contrary, following neutrophil depletion with anti-Ly6G antibody, neutrophil infiltration into lung tissue was greatly reduced, while the recruitment of other types of immune cells was not much affected, except for monocyte recruitment that was slightly increased at day 5 (Figure 9A).

To study the effect of neutrophil depletion on TDM-induced inflammation leading to granuloma formation, the development of inflammatory foci with infiltrated immune cells was examined. Lung sections prepared from Ly6G mAb-treated mice and control mice challenged with TDM were H&E stained. Two days after the TDM treatment, lungs from control mice had higher numbers of foci with accumulated neutrophils and macrophages than did lungs from Ly6G mAb-treated mice. However, there were no discernible differences in granuloma formation 5 days after the TDM treatment (Figure 9B and 9C). Immunohistochemical analysis identified Ly6G^+^ neutrophils around the vessels and in the granulomas of the control lung tissues, but these cells were absent in the lung tissues from Ly6G mAb-treated mice (Figure 9C). These data indicate that infiltrated neutrophils may affect lung inflammation during the early stage following TDM administration. In addition, Ly6G mAb treated mice had reduced IL-6 and MCP-1 protein concentrations in whole-lung homogenates 2 days after TDM administration (Figure 9D). TNFα concentrations were lower in serum from Ly6G mAb treated mice than in that from control mice (Figure 9E), even though TNFα concentrations were not altered in whole-lung homogenates. Because the number of monocytes recruited to the TDM-challenged lung is higher than that of neutrophils, TNFα produced by the monocytes may compensate the loss from the depleted neutrophils. Although these neutrophil depletion conditions were not sufficient to prevent granuloma formation, the reduction of IL-6 and MCP-1 production during the early stage of TDM-induced lung inflammation indicates that mycobacterial TDM can activate neutrophils to produce key inflammatory factors that contribute to the amplification of acute lung inflammation.

## Discussion

Here, we report several interesting findings on the role of Mincle signaling on neutrophils during the early stage of mycobacterial TDM-induced inflammation. First, neutrophils were recruited to TDM-challenged sites through Mincle signaling and contributed to the innate immune response by producing proinflammatory cytokines/chemokines. Second, TDM-induced Mincle signaling promoted neutrophil adhesion by up-regulating CD11b/CD18 surface expression through Src, Syk, and MEK signaling. Third, coactivation of TLR2 potentiated TDM-activated Mincle signaling by up-regulating Mincle surface expression on neutrophils. Fourth, in a murine model, Mincle activity was required for defense against Mtb infection, and the lack of neutrophils during the TDM-induced inflammation caused defects in early immune responses. These properties of Mincle signaling during TDM-induced lung inflammation may provide valuable clues for determining the pathological role of neutrophils during Mtb-induced diseases.

Mycobacterial cell walls contain diverse PAMPs (such as phosphatidylinositol dimannoside, phosphatidylinositol hexamannoside, and TDM) that can trigger granuloma formation [35]–[38]. Among these components, TDM is a virulence factor that can mimic the pathogenesis of pulmonary tuberculosis, including excessive proinflammatory cytokine production, granulomatous responses, weight loss, and caseous necrosis when administered as a monolayer or part of an oil-in-water emulsion [4], [39]–[41]. TDM-deprived Mtb remains viable, but fails to induce accelerating infections when injected into mice. Removal of TDM from the surface of mycobacteria reduces their ability to survive in macrophages and in the lungs and spleens of mice [42]–[44]. A loss of virulence is also correlated with a disruption of the ability of Mtb to assemble excess TDM [45], [46]. Moreover, mutations that alter the structure of mycolic acids on TDM can suppress or increase the virulence of Mtb. For example, a *pcaA* mutant form of Mtb that is unable to modify the cis-cyclopropane of mycolic acid on TDM, has attenuated virulence and invokes less severe immunopathology than WT Mtb [47]. However, a *cmaA2* mutant Mtb that lacks trans-cycloproponation of the mycolic acids on TDM is hypervirulent while inducing larger granulomas than does WT Mtb [48]. Therefore, TDM may be a key driver of Mtb-induced tuberculosis. Thus, the regulatory mechanisms of TDM-induced immune responses will provide fundamental clues in the understanding of Mtb-induced pathogenesis. However, TDM-induced lung inflammation delivered through intravenously injection may be different from Mtb infection through aerosol-inhalation because additional ligands of mycobacteria activate diverse immune signals directly on the alveolar cells, and the additional immune evading mechanisms of live mycobacteria can induce complex responses *in vivo*.

Neutrophils play very important roles in innate immune responses against bacterial infections. However, the role of neutrophils in Mtb infection remains controversial. Pedrosa and colleagues indicated that the absence of neutrophils significantly impaired the defense against the initial stages of mycobacterial infections [49]. Additionally, LPS-induced transient neutrophil recruitment prevents early mycobacterial infections [50]. On the other hand, Seiler and colleagues showed that neutrophils are not involved in the early control of Mtb infections [51]. Some of these differences may be due to the specificity of the antibodies used for neutrophil depletion or the mycobacteria strains used. Although our neutrophil depletion experiments in mice showed no major defects in granuloma formation, the initiation of immune cell recruitment appears to have been retarded with defects in IL-6 and MCP-1 production. Considering the higher bacterial load in the Mincle-mutant mice and the presence of macrophage-driven granulomas equivalent to those found in WT mice, Mincle–mediated immune responses may be required to clear Mtb infections during the initial phase of inflammation. However, the use of neutrophil-specific Mincle knockout mice is needed to confirm the distinct function of neutrophils against Mtb infection.

Although we demonstrated TDM-Mincle mediated inflammatory responses in neutrophils, Mincle signaling is also activated by endogenous protein, SAP130 from dead cells [17]. It is known that TDM administration induces necrosis of host cells, and necrotic bodies are indeed existed in the center of granulomas [1], [52]. Therefore, it is possible that both TDM from mycobacterial PAMP and endogenous ligand from dead cells may contribute to induce inflammatory responses through Mincle signaling after TDM administration in vivo.

In this report, we show that the Mincle downstream molecules Srk, Syk, and MAPK/ERK kinases play a key role in neutrophil adherence to TDM. Our results support and extend previous findings demonstrating that the Mincle signaling pathway produces proinflammatory cytokines via the FcRγ-Syk pathway in macrophages [13]. One important difference between macrophages and neutrophils is that neutrophils induce up-regulation of cell-surface CD11b/CD18 in response to TDM. Generally, CD11b/CD18 is not constitutively in its active form. However, when an immune response stimulates neutrophils, the CD11b/CD18 activity is controlled by signaling through immune receptors. This ‘inside-out’ signaling converts integrins from inactive to active forms [26], [53]. Fc receptors for IgG induce CD11b/CD18 activation via inside-out signaling [54], and the up-regulation of CD11b/CD18 by activated FcγRIIA and FcγRIIIB is mediated through Src and Syk kinase activation [55], [56]. These CD11b/CD18 conformational changes result in increased ligand binding affinities and clustering of integrins on the membrane leading to cell attachment. Our work identified that robust neutrophil adherence is also induced by Mincle-mediated inside-out CD11b/CD18 surface expression. Mincle-induced CD11b/CD18 clustering may also affect leukocyte rolling and transmigration on the vascular endothelium to initiate innate immune responses following Mtb infection.

Although both Mincle and TLR activation induced actin remodeling, these receptors likely mediate different cellular reorganizations. TLR ligation leads to the coordinated redeployment of actin to provide fuel for endocytosis by dendritic cells [57]. However, active F-actin polymerization induced by TDM/Mincle signaling promoted cell adhesion by inducing high levels of CD11b/CD18 surface expression. Therefore, Mincle signaling on neutrophils plays a unique role in the remodeling of the actin cytoskeleton that is required for cell adhesion.

Several studies have shown that synergistic proinflammatory cytokine production and TLR (TLR2 and TLR4) signaling is necessary for the coactivation of CLRs, such as Dectin-1 and MICL/DCAL-2 in macrophages and DCs [58]–[60]. In this report, we describe the synergistic interactions between the Mincle and TLR2 signaling pathways in neutrophils. Whereas TDM stimulation resulted in the slow and steady induction of diverse neutrophil responses (CD11b/CD18 surface expression, cell adhesion, ROS release, and TNFα production), co-stimulation with TDM and Pam3CSK4 rapidly and strongly up-regulated these responses. One possible explanation is that the large induction of Mincle by TLR ligands may result in additional TDM signaling. A similar mechanism was used with epithelial cells, and the level of TLR2 on unstimulated epithelial cells was markedly enhanced in response to invading microbes [61], [62]. Mincle was also up-regulated on macrophages after exposure to various stimuli and cellular stresses, such as LPS, TNFα, and IL-6 [63]. Our studies confirmed that Mincle expression on neutrophil surfaces was highly up-regulated following Pam3CSK4 stimulation. Therefore, the synergistic inflammatory responses of neutrophils likely resulted from the positive enforcement of Mincle expression by TLR activation.

During the initiation of Mtb-induced granuloma formation, it is thought that TNFα production by infected macrophages drives the recruitment of neutrophils, which in turn produce diverse cytokines and chemokines. Consequently, a large number of lymphocytes are recruited to the site of infection. In murine models of Mtb infection, TNFα is essential for the formation of granulomas [64], [65]. However, the early recruitment of neutrophils prior to the majority of other cell types and the dramatic induction of immune responses by neutrophils following coactivation of Mincle and TLR2, indicates that neutrophils may be recruited to the site of Mtb infection by direct recognition of TDM. In particular, IL-6 and MCP-1 expressions in infected lung were strongly correlated with the early recruitment of neutrophils. Despite of the strong Mincle-mediated responses of neutrophils at the initiation of inflammation, the redundant function of macrophages appears to be sufficient for the granuloma maturation. Therefore, neutrophils may play a major role in the initiation of immune responses against Mtb infection rather than working in the granuloma formation after being passively recruited by activated immune cells.

Although serum TNFα levels were also decreased in neutrophil-depleted mice during Mtb infection conditions, lung TNFα expression levels were not significantly affected by the depletion of neutrophils. Contrary to the early expression pattern of IL-6 and MCP-1, TNFα expression was extended for a few more days, thus overlapping with monocyte recruitment to the infection sites, suggesting that monocytes may contribute to TNFα production as well. Therefore, the large number of recruited monocytes may compensate for the loss of TNFα expression caused by neutrophil depletion at the localized infection site.

In summary, our study provides evidence that the Mincle signaling pathway in neutrophils promotes diverse neutrophil activation responses (cell adherence, CD11b/CD18 surface expression, ROS release, and TNFα production) in response to mycobacterial TDM. These immune responses to Mtb via Mincle signaling were enhanced by coactivation of TLR2. Furthermore, neutrophils produced proinflammatory cytokines/chemokines during the early stage of TDM-induced pulmonary inflammation. These findings suggest that the Mincle pathway in neutrophils is able to modulate lung inflammation.

## Materials and Methods

### Ethics statement

All animal experiments were performed in accordance with the Korean Food and Drug Administration (KFDA) guidelines. Protocols were approved by the Institutional Animal Care and Use Committees of the Laboratory Animal Research Center at Yonsei University (Permit Number: 2007-0001).

### Animals

C57BL/6 mice, aged 6–7 weeks, were purchased from Orient Bio (Gwangju, Gyeonggi, Korea). Mincle^−/−^ mice (Clec4e^MNA^) were kindly provided by the Consortium for Functional Glycomics (http://www.functionalglycomics.org) and were backcrossed for nine generations to the C57BL/6 background. MyD88-deficient mice (C57BL/6 background) were purchased from Oriental BioService (Kyoto, Japan). All mice were maintained in the specific pathogen-free facility of the Laboratory Animal Research Center at Yonsei University.

### Induction of pulmonary granulomas through administration of TDM

Because a proper orientation of TDM on the mycobacterial surface is required for its inflammatory and immunoregulatory properties [1], TDM was prepared as a water-in-oil emulsion to potentiate its immunostimulatory activity as shown previously [5], [40]. Mice (9–11 weeks old) were injected intravenously in the tail vein with 100 µl emulsion containing 100 µg TDM (Sigma, St. Louis, MO). Mice were sacrificed at days 1, 2, 5, and 7 post-TDM challenge. Lungs were weighed and fixed in 10% formaldehyde for H&E staining and immunohistochemistry. Lung sections were frozen for quantitative reverse transcription-polymerase chain reaction (RT-PCR). A previously-described lung weight index [66] was used. Neutrophil depletion was achieved by intravenous tail injection of 200 µg anti-Ly6G (1A8) mAb or isotype control antibodies 1 day prior to TDM administration. Purified 1A8 mAb for neutrophil depletion was purchased from BioXcell (West Lebanon, NH). Quantitation of granuloma areas from at least five randomly-selected fields from each slide of 3 independent experiments was performed using Adobe Photoshop software (Adobe Systems, San Jose, CA). Results are expressed as relative granuloma area per fixed field of view at a magnification of ×40.

### Delivery of TDM-bead-bearing matrices

An *in vivo* murine granuloma model was prepared as described previously [52]. Briefly, 2×10^3^ 90-µm polystyrene microspheres (Polysciences, Inc., Warrington, PA) were coated with 15 µg TDM. The TDM-coated beads were mixed with bone marrow-derived macrophages (BMMs; 1×10^7^ cells/ml) in 300 µl ice-cold growth factor-reduced Matrigel (BD Bioscience, Franklin Lakes, NJ). Matrices were mixed with the indicated BMM genotypes and subcutaneously injected at each site in the flank of WT or Mincle^−/−^ mice. Three mice were injected with TDM-coated bead matrices. The experiment was repeated more than three times. Matrices were harvested at 20 h post-inoculation and fixed in 10% formaldehyde for H&E staining and immunohistochemical analysis. Neutrophils (Ly6G^+^) or macrophages (F4/80^+^) surrounding the TDM-coated beads were counted under a light microscope (Nikon ECLIPSE 80i) on at least five randomly-selected fields.

### Mtb infection and bacterial counts

Mice were challenged by aerosol exposure with WT virulent Mtb Erdman using an inhalation device (Glas-Col, Terre Haute, IN) calibrated to deliver approximately 100 bacteria into the lungs. Five mice per group were sacrificed on days 14 and 60 post-challenge, and bacteria in lung homogenates were counted. Numbers of viable bacteria in lungs were determined by plating serial dilutions of whole organ homogenates on Middlebrook 7H11 agar (Difco, Detroit, MI). Colonies were counted after 3–4 weeks of incubation at 37°C.

### Preparation of bone marrow neutrophils and neutrophil assay conditions

Murine bone marrow neutrophils were isolated using Percoll (GE Healthcare, Little Chalfont, UK) density gradient centrifugation and hypotonic lysis of red blood cells. Murine bone marrow cells were layered on top of a 53/63/76% three-layer Percoll gradient following removal of red blood cells. Following centrifugation, mature neutrophils were recovered at the interface of the 63% and 76% fractions and were >90% pure and >95% viable in the neutrophil-rich fraction as determined by Diff-Quick staining and trypan blue exclusion, respectively. Neutrophils were suspended in RPMI-1640 medium with 5% fetal calf serum (Invitrogen, Grand Island, NY).

### F-actin polymerization

Neutrophils were pre-incubated with the indicated inhibitors in RPMI-1640 medium with 5% fetal bovine serum for 30 min at 37°C. Cells were stimulated for 40 min at 37°C with TDM (25 µg/ml, Sigma), ultrapure LPS (1 µg/ml, InvivoGen, San Diego, CA), Pam3CSK4 (1 µg/ml, InvivoGen), or *E. coli*-peptidoglycan (0.2 mg/ml, Sigma) on coverslips. After stimulation, cells were fixed with 4% paraformaldehyde, permeabilized with BD Perm/Wash Buffer (BD Biosciences), and stained with Alexa568-phalloidin (Molecular Probes, Eugene, Oregon). Microscopic analysis was performed using an Olympus DP-40 microscope. Mean fluorescence intensities were determined from the measurement of individual cells' fluorescence intensities. More than 20 cells from at least five randomly-selected fields from each slide were analyzed.

Alternatively, measurement of actin polymerization was done by a flow-cytometry based phalloidin-binding assay [67]. After stimulation with TDM for 18 h, neutrophils were fixed in 2% para-formaldehyde for 15 min at 37°C. Cells were then stained with fluorescein phalloidin (Invitrogen) in permeabilization buffer (BD biosciences), and analyzed by flow cytometry.

### Adhesion assay

Neutrophils (3×10^5^ cells/well) were allowed to adhere to 48-well plates in the presence or absence of TDM (25 µg/ml) with the indicated inhibitors or mAb M1/70 (0.5 mg/ml, BD Biosciences) for 20 h at 37°C. In some experiments, cells were stimulated with TDM and TNFα (50 ng/ml) for 1 h. After stimulation, the non-adherent cells were washed away. Adherent neutrophils were quantified with a myeloperoxidase (MPO) assay. MPO activities were determined through H_2_O_2_-dependent oxidation of 3,3′,5,5′-tetramethylbenzine (Sigma). Adherence was expressed as the ratio of adherent neutrophil MPO activity to that of total neutrophils.

Alternatively, neutrophils were incubated with 5 µM calcein-acetoxymethyl ester at 37°C for 30 min. Thereafter, the cells were washed, resuspended in RPMI containing 10% FCS media, and allowed to adhere to TDM-coated 48-well plates for 6 h. The cells were washed three times and the nonadherent cells were removed. The fluorescence in each well measured by a fluorescence microplate reader both before and after washing, and the percentage of adherent cells was calculated.

### Flow cytometric analysis

Lungs were recovered, weighed, incubated in 2 mg/ml collagenase D (Roche, Basel, Switzerland) and 40 U/ml DNase I (Roche) solution, and dispersed by passage through a 70 µm mesh. After lysis of red blood cells, viable cells were counted. For immunophenotyping, cells were incubated with fluorescence-conjugated antibodies. Antibodies (BD Pharmingen, San Jose, CA) used were against Gr-1 (RB6-8C5), CD11b (M1/70), Ly6G (1A8), CD3ε (145-2C11), and CD19 (1D3). Cells positive for propidium iodide were excluded by gating prior to collecting at least 10,000 events. To quantify CD11b, CD18, and CD62L expression levels on the surface of neutrophils, cells were incubated with mAbs (1∶50) labeled with fluorescein isothiocyanate (FITC) or phycoerythrin (PE). Stained neutrophils were washed with staining buffer, and the fluorescence of 3×10^5^ cells per sample was analyzed on a FACS Calibur (BD Biosciences). Results were expressed as fluorescence intensities on a logarithmic scale. Anti-mouse CD11b (M1/70), CD18 (C71/16), CD62L (MEL-14), and isotype control antibodies were obtained from BD Pharmingen. To quantify Mincle expression, cells were incubated in a blocking solution containing mouse serum. Blocked cells were incubated with an anti-mouse Mincle antibody (4A9, MBL International, Woburn, MA).

### ROS assay

ROS were analyzed with H_2_DCFDA assays. For the detection of ROS production following TDM stimulation, neutrophils (1×10^5^ cells/well in 96-well plates) were pre-incubated with 10 µM H_2_DCFDA (Molecular Probes) in PBS for 30 min allowing the dye to enter the cells. Cells were washed with PBS prior to stimulation with TDM for 6 h. Plates were analyzed at 495 nm/520 nm on a microplate reader. Intracellular ROS levels were detected by dihydrorhodamine123 (DHR123). Neutrophils (5×10^5^ cells) were incubated in 5 µM DHR123 for 30 min prior to TDM and Pam3CSK4 stimulation. ROS expression levels were analyzed by flow cytometry.

### Measurement of cytokines

Cytokine levels in neutrophil culture supernatants and mouse sera were assayed using Cytometric Bead Arrays (CBA) from BD Biosciences according to the manufacturer's instructions (Mouse Inflammation Kit: TNFα, IL-6, MCP-1, IL-10, IL-12p40, and IFNγ).

### Western blotting

Neutrophils were stimulated with TNFα (50 ng/ml) and TDM (25 µg/ml) for 30 min at 37°C. Cell lysates were prepared as described previously [68]. Anti-phospho-Erk (Thr202/Tyr204), -phospho-p38, -phospho-Jnk, -Erk, -p38, and -Jnk antibodies were obtained from Cell Signaling Technology (Beverly, MA).

### RNA extraction and quantitative RT-PCR

Lung tissues from TDM-challenged and control mice were homogenized and frozen in Trizol Reagent (Invitrogen). Total RNA was extracted according to the manufacturer's protocol. cDNA was synthesized using SuperScript II Reverse Transcriptase (Invitrogen). Quantitative real-time RT-PCR analysis of the cDNA was performed using a LightCycler 480 Real-Time PCR System (Roche) with a SYBR Green dye. The relative mRNA expression of each gene was determined using the ΔΔCt calculation method with Hprt as the internal control gene. The following primer sequences were used: 5′-CTGAGGCGGCGAGGGAGAG-3′ and 5′-AAGCGGTCTGAGGAGGAAGCC-3′ for Hprt; 5′-AGTGAGGCATCAGGTTCAGTCAAG-3′ and 5′-GACCAGGTCAAGGTTGTCGTAGAG-3′ for Mincle; 5′-CCAAGACCATCCAATTCATC-3′ and 5′-CCACAAACTGATATGCTTAGG-3′ for IL-6; and 5′-TTCCACAACCACCTCAAGCACTTC-3′ and 5′-TTAAGGCATCACAGTCCGAGTCAC-3′ for MCP-1.

### Statistical analysis

Data are presented as means ± standard error of the mean (SEM). Statistical comparisons between groups were performed using one-way analysis of variance followed by Student's *t*-tests.

## Supporting Information

Figure S1 **Surface expression of Mincle on neutrophils was elevated by TDM stimulation.** Surface expression of Mincle was determined by flow cytometry. Neutrophils from wild-type (WT) and Mincle^−/−^ mice were stimulated with 25 µg/ml trehalose dimycolate (TDM) for 18 h and analyzed by flow cytometry. Level of Mincle surface expression was quantified by mean fluorescence intensity (MFI). Statistical significance: *p<0.05 and **p<0.01. Data are expressed as means ± SEM from three independent experiments.
(TIF)

Figure S2 **Neutrophil adhesion was increased by TDM stimulation.** Suspended wild-type (WT) and Mincle^−/−^ neutrophils were preincubated with calcein-acetoxymethyl ester at 37°C for 30 min. Then, the cells were incubated for 6 h on trehalose dimycolate (TDM)-coated plate. Nonadherent cells were removed, and then the adhered neutrophils were imaged by fluorescent microscopy. Original magnification was 200×. And fluorescence of each well was measured by using a fluorescence microplate reader (means ± SEM). Statistical significance: **p<0.01 and ***p<0.001. Data are expressed as means ± SEM from three independent experiments.
(TIF)

Figure S3 **Actin polymerization was induced in response to TDM.** Wild-type (WT) and Mincle^−/−^ neutrophils were stimulated with trehalose dimycolate (TDM) for 18 h, and actin polymerization was measured by phalloidin staining and flow cytometry analysis. Results are given as mean ± SEM from three independent experiments. Statistical significance: *p<0.05.
(TIF)

Figure S4 **Cell adhesion was synergistically increased following co-stimulation with TDM/Pam3CSK4.** Wild-type (WT), Mincle^−/−^, MyD88^−/−^, and Mincle^−/−^MyD88^−/−^ (DKO) bone marrow neutrophils were preincubated with calcein-acetoxymethyl ester at 37°C for 30 min. Then, the cells were stimulated with Pam3CSK4 (10 ng/ml) and/or trehalose dimycolate (TDM, 25 µg/ml) for 6 h. The adhered neutrophils were imaged by fluorescent microscopy. Original magnification was 200×. And fluorescence of each well was measured by using a fluorescence microplate reader (means ± SEM). Statistical significance: **p<0.01 and ***p<0.001. Data are expressed as means ± SEM from three independent experiments.
(TIF)

## References

[1] Hunter RL, Olsen MR, Jagannath C, Actor JK (2006). Multiple roles of cord factor in the pathogenesis of primary, secondary, and cavitary tuberculosis, including a revised description of the pathology of secondary disease.. Ann Clin Lab Sci.

[2] Russell DG (2006). Who puts the tubercle in tuberculosis?. Nat Rev Microbiol.

[3] Russell DG, Barry CE, Flynn JL (2010). Tuberculosis: What We Don't Know Can, and Does, Hurt Us.. Science.

[4] Hunter RL, Olsen M, Jagannath C, Actor JK (2006). Trehalose 6,6′-dimycolate and lipid in the pathogenesis of caseating granulomas of tuberculosis in mice.. Am J Pathol.

[5] Perez RL, Roman J, Roser S, Little C, Olsen M (2000). Cytokine message and protein expression during lung granuloma formation and resolution induced by the mycobacterial cord factor trehalose-6,6′-dimycolate.. J Interferon Cytokine Res.

[6] Janeway CA, Medzhitov R (2002). Innate immune recognition.. Annu Rev Immunol.

[7] Robinson MJ, Sancho D, Slack EC, LeibundGut-Landmann S, Reis e Sousa C (2006). Myeloid C-type lectins in innate immunity.. Nat Immunol.

[8] Ishikawa E, Ishikawa T, Morita YS, Toyonaga K, Yamada H (2009). Direct recognition of the mycobacterial glycolipid, trehalose dimycolate, by C-type lectin Mincle.. J Exp Med.

[9] Schoenen H, Bodendorfer B, Hitchens K, Manzanero S, Werninghaus K (2010). Cutting Edge: Mincle is essential for recognition and adjuvanticity of the mycobacterial cord factor and its synthetic analog trehalose-dibehenate.. J Immunol.

[10] Wells CA, Salvage JAJ, Li X, Hitchens K, Butcher S (2008). The macrophage-inducible C-type lectin, mincle, is an essential component of the innate immune response to candida albicans.. J Immunol.

[11] Yamasaki S, Matsumoto M, Takeuchi O, Matsuzawa T, Ishikawa E (2009). C-type lectin Mincle is an activating receptor for pathogenic fungus, Malassezia.. Proc Natl Acad Sci U S A.

[12] da Glória Sousa M, Reid Delyth M, Schweighoffer E, Tybulewicz V, Ruland J (2011). Restoration of Pattern Recognition Receptor Costimulation to Treat Chromoblastomycosis, a Chronic Fungal Infection of the Skin.. Cell Host Microbe.

[13] Yamasaki S, Ishikawa E, Sakuma M, Hara H, Ogata K (2008). Mincle is an ITAM-coupled activating receptor that senses damaged cells.. Nat Immunol.

[14] Werninghaus K, Babiak A, Gross O, Holscher C, Dietrich H (2009). Adjuvanticity of a synthetic cord factor analogue for subunit Mycobacterium tuberculosis vaccination requires FcR -Syk-Card9-dependent innate immune activation.. J Exp Med.

[15] Welsh KJ, Abbott AN, Hwang SA, Indrigo J, Armitige LY (2008). A role for tumour necrosis factor-, complement C5 and interleukin-6 in the initiation and development of the mycobacterial cord factor trehalose 6,6′-dimycolate induced granulomatous response.. Microbiology.

[16] Eum SY, Kong JH, Hong MS, Lee YJ, Kim JH (2010). Neutrophils are the predominant infected phagocytic cells in the airways of patients with active pulmonary TB.. Chest.

[17] Berry MP, Graham CM, McNab FW, Xu Z, Bloch SA (2010). An interferon-inducible neutrophil-driven blood transcriptional signature in human tuberculosis.. Nature.

[18] Aleman M, Beigier-Bompadre M, Borghetti C, de la Barrera S, Abbate E (2001). Activation of peripheral blood neutrophils from patients with active advanced tuberculosis.. Clin Immunol.

[19] Kasahara K, Sato I, Ogura K, Takeuchi H, Kobayashi K (1998). Expression of chemokines and induction of rapid cell death in human blood neutrophils by Mycobacterium tuberculosis.. J Infect Dis.

[20] Perez RL, Roman J, Staton GW, Hunter RL (1994). Extravascular coagulation and fibrinolysis in murine lung inflammation induced by the mycobacterial cord factor trehalose-6,6′-dimycolate.. Am J Respir Crit Care Med.

[21] Greenblatt MB, Aliprantis A, Hu B, Glimcher LH (2010). Calcineurin regulates innate antifungal immunity in neutrophils.. J Exp Med.

[22] Kerrigan AM, Dennehy KM, Mourao-Sa D, Faro-Trindade I, Willment JA (2009). CLEC-2 is a phagocytic activation receptor expressed on murine peripheral blood neutrophils.. J Immunol.

[23] Kim MJ, Wainwright HC, Locketz M, Bekker LG, Walther GB (2010). Caseation of human tuberculosis granulomas correlates with elevated host lipid metabolism.. EMBO Mol Med.

[24] Lauterbach M, O'Donnell P, Asano K, Mayadas TN (2008). Role of TNF priming and adhesion molecules in neutrophil recruitment to intravascular immune complexes.. J Leukoc Biol.

[25] Mócsai A, Abram CL, Jakus Z, Hu Y, Lanier LL (2006). Integrin signaling in neutrophils and macrophages uses adaptors containing immunoreceptor tyrosine-based activation motifs.. Nat Immunol.

[26] Evans R, Patzak I, Svensson L, De Filippo K, Jones K (2009). Integrins in immunity.. J Cell Sci.

[27] Tellier E, Canault M, Rebsomen L, Bonardo B, Juhan-Vague I (2006). The shedding activity of ADAM17 is sequestered in lipid rafts.. Exp Cell Res.

[28] Walcheck B, Kahn J, Fisher JM, Wang BB, Fisk RS (1996). Neutrophil rolling altered by inhibition of L-selectin shedding in vitro.. Nature.

[29] Wang Q, Doerschuk CM (2001). The p38 mitogen-activated protein kinase mediates cytoskeletal remodeling in pulmonary microvascular endothelial cells upon intracellular adhesion molecule-1 ligation.. J Immunol.

[30] Pichon S, Bryckaert M, Berrou E (2004). Control of actin dynamics by p38 MAP kinase - Hsp27 distribution in the lamellipodium of smooth muscle cells.. J Cell Sci.

[31] Kleinnijenhuis J, Oosting M, Joosten LA, Netea MG, Van Crevel R (2011). Innate Immune Recognition of Mycobacterium tuberculosis.. Clin Dev Immunol.

[32] Reiling N, Holscher C, Fehrenbach A, Kroger S, Kirschning CJ (2002). Cutting edge: Toll-like receptor (TLR)2- and TLR4-mediated pathogen recognition in resistance to airborne infection with Mycobacterium tuberculosis.. J Immunol.

[33] Drennan MB, Nicolle D, Quesniaux VJ, Jacobs M, Allie N (2004). Toll-like receptor 2-deficient mice succumb to Mycobacterium tuberculosis infection.. Am J Pathol.

[34] Daley JM, Thomay AA, Connolly MD, Reichner JS, Albina JE (2008). Use of Ly6G-specific monoclonal antibody to deplete neutrophils in mice.. J Leukoc Biol.

[35] Bekierkunst A (1968). Acute granulomatous response produced in mice by trehalose-6,6-dimycolate.. J Bacteriol.

[36] Gilleron M, Ronet C, Mempel M, Monsarrat B, Gachelin G (2001). Acylation state of the phosphatidylinositol mannosides from Mycobacterium bovis bacillus Calmette Guerin and ability to induce granuloma and recruit natural killer T cells.. J Biol Chem.

[37] Apostolou I, Takahama Y, Belmant C, Kawano T, Huerre M (1999). Murine natural killer T(NKT) cells [correction of natural killer cells] contribute to the granulomatous reaction caused by mycobacterial cell walls.. Proc Natl Acad Sci U S A.

[38] Yamagami H, Matsumoto T, Fujiwara N, Arakawa T, Kaneda K (2001). Trehalose 6,6′-dimycolate (cord factor) of Mycobacterium tuberculosis induces foreign-body- and hypersensitivity-type granulomas in mice.. Infect Immun.

[39] Bekierkunst A, Levij IS, Yarkoni E, Vilkas E, Adam A (1969). Granuloma formation induced in mice by chemically defined mycobacterial fractions.. J Bacteriol.

[40] Yarkoni E, Rapp HJ (1977). Granuloma formation in lungs of mice after intravenous administration of emulsified trehalose-6,6′-dimycolate (cord factor): reaction intensity depends on size distribution of the oil droplets.. Infect Immun.

[41] Geisel RE, Sakamoto K, Russell DG, Rhoades ER (2005). In vivo activity of released cell wall lipids of Mycobacterium bovis bacillus Calmette-Guerin is due principally to trehalose mycolates.. J Immunol.

[42] Indrigo J (2003). Cord factor trehalose 6,6′-dimycolate (TDM) mediates trafficking events during mycobacterial infection of murine macrophages.. Microbiology.

[43] Silva CL, Ekizlerian SM, Fazioli RA (1985). Role of cord factor in the modulation of infection caused by mycobacteria.. Am J Pathol.

[44] Lima VMF, Bonato VLD, Lima KM, Dos Santos SA, Dos Santos RR (2001). Role of Trehalose Dimycolate in Recruitment of Cells and Modulation of Production of Cytokines and NO in Tuberculosis.. Infect Immun.

[45] Hunter RL, Venkataprasad N, Olsen MR (2006). The role of trehalose dimycolate (cord factor) on morphology of virulent M. tuberculosis in vitro.. Tuberculosis (Edinb).

[46] Armitige LY, Jagannath C, Wanger AR, Norris SJ (2000). Disruption of the genes encoding antigen 85A and antigen 85B of Mycobacterium tuberculosis H37Rv: effect on growth in culture and in macrophages.. Infect Immun.

[47] Rao V, Fujiwara N, Porcelli SA, Glickman MS (2005). Mycobacterium tuberculosis controls host innate immune activation through cyclopropane modification of a glycolipid effector molecule.. J Exp Med.

[48] Rao V, Gao F, Chen B, Jacobs WR, Glickman MS (2006). Trans-cyclopropanation of mycolic acids on trehalose dimycolate suppresses Mycobacterium tuberculosis-induced inflammation and virulence.. J Clin Invest.

[49] Pedrosa J, Saunders BM, Appelberg R, Orme IM, Silva MT (2000). Neutrophils play a protective nonphagocytic role in systemic Mycobacterium tuberculosis infection of mice.. Infect Immun.

[50] Sugawara I, Udagawa T, Yamada H (2004). Rat neutrophils prevent the development of tuberculosis.. Infect Immun.

[51] Seiler P, Aichele P, Raupach B, Odermatt B, Steinhoff U (2000). Rapid neutrophil response controls fast-replicating intracellular bacteria but not slow-replicating Mycobacterium tuberculosis.. J Infect Dis.

[52] Sakamoto K, Geisel RE, Kim MJ, Wyatt BT, Sellers LB (2009). Fibrinogen Regulates the Cytotoxicity of Mycobacterial Trehalose Dimycolate but Is Not Required for Cell Recruitment, Cytokine Response, or Control of Mycobacterial Infection.. Infect Immun.

[53] Abram CL, Lowell CA (2009). The ins and outs of leukocyte integrin signaling.. Annu Rev Immunol.

[54] Ortiz-Stern A, Rosales C (2003). Cross-talk between Fc receptors and integrins.. Immunol Lett.

[55] Ortiz-Stern A, Rosales C (2005). Fc gammaRIIIB stimulation promotes beta1 integrin activation in human neutrophils.. J Leukoc Biol.

[56] Kocher M, Siegel ME, Edberg JC, Kimberly RP (1997). Cross-linking of Fc gamma receptor IIa and Fc gamma receptor IIIb induces different proadhesive phenotypes on human neutrophils.. J Immunol.

[57] West MA, Wallin RP, Matthews SP, Svensson HG, Zaru R (2004). Enhanced dendritic cell antigen capture via toll-like receptor-induced actin remodeling.. Science.

[58] Dennehy KM, Ferwerda G, Faro-Trindade I, Pyz E, Willment JA (2008). Syk kinase is required for collaborative cytokine production induced through Dectin-1 and Toll-like receptors.. Eur J Immunol.

[59] Gantner BN, Simmons RM, Canavera SJ, Akira S, Underhill DM (2003). Collaborative induction of inflammatory responses by dectin-1 and Toll-like receptor 2.. J Exp Med.

[60] Chen CH, Floyd H, Olson NE, Magaletti D, Li C (2006). Dendritic-cell-associated C-type lectin 2 (DCAL-2) alters dendritic-cell maturation and cytokine production.. Blood.

[61] Sakai A, Han J, Cato AC, Akira S, Li JD (2004). Glucocorticoids synergize with IL-1beta to induce TLR2 expression via MAP Kinase Phosphatase-1-dependent dual Inhibition of MAPK JNK and p38 in epithelial cells.. BMC Mol Biol.

[62] Shuto T, Imasato A, Jono H, Sakai A, Xu H (2002). Glucocorticoids synergistically enhance nontypeable Haemophilus influenzae-induced Toll-like receptor 2 expression via a negative cross-talk with p38 MAP kinase.. J Biol Chem.

[63] Matsumoto M, Tanaka T, Kaisho T, Sanjo H, Copeland NG (1999). A novel LPS-inducible C-type lectin is a transcriptional target of NF-IL6 in macrophages.. J Immunol.

[64] Flynn JL, Goldstein MM, Chan J, Triebold KJ, Pfeffer K (1995). Tumor necrosis factor-α is required in the protective immune response against mycobacterium tuberculosis in mice.. Immunity.

[65] Bean AGD, Roach DR, Briscoe H, France MP, Korner H (1999). Structural deficiencies in granuloma formation in TNF gene-targeted mice underlie the heightened susceptibility to aerosol Mycobacterium tuberculosis infection, which is not compensated for by lymphotoxin.. J Immunol.

[66] Guidry TV, Olsen M, Kil KS, Hunter RL, Geng YJ (2004). Failure of CD1D^−/−^ mice to elicit hypersensitive granulomas to mycobacterial cord factor trehalose 6,6′-dimycolate.. J Interferon Cytokine Res.

[67] Van Ziffle JA, Lowell CA (2009). Neutrophil-specific deletion of Syk kinase results in reduced host defense to bacterial infection.. Blood.

[68] Mócsai A, Zhou M, Meng F, Tybulewicz VL, Lowell CA (2002). Syk is required for integrin signaling in neutrophils.. Immunity.

