# Supplementary figures and images for: Neutrophils Promote Mycobacterial Trehalose Dimycolate-Induced Lung Inflammation via the Mincle Pathway
Source: PLoS Pathog. 2012 Apr 5;8(4):e1002614. doi: 10.1371/journal.ppat.1002614 (PMC3320589; doi:10.1371/journal.ppat.1002614)

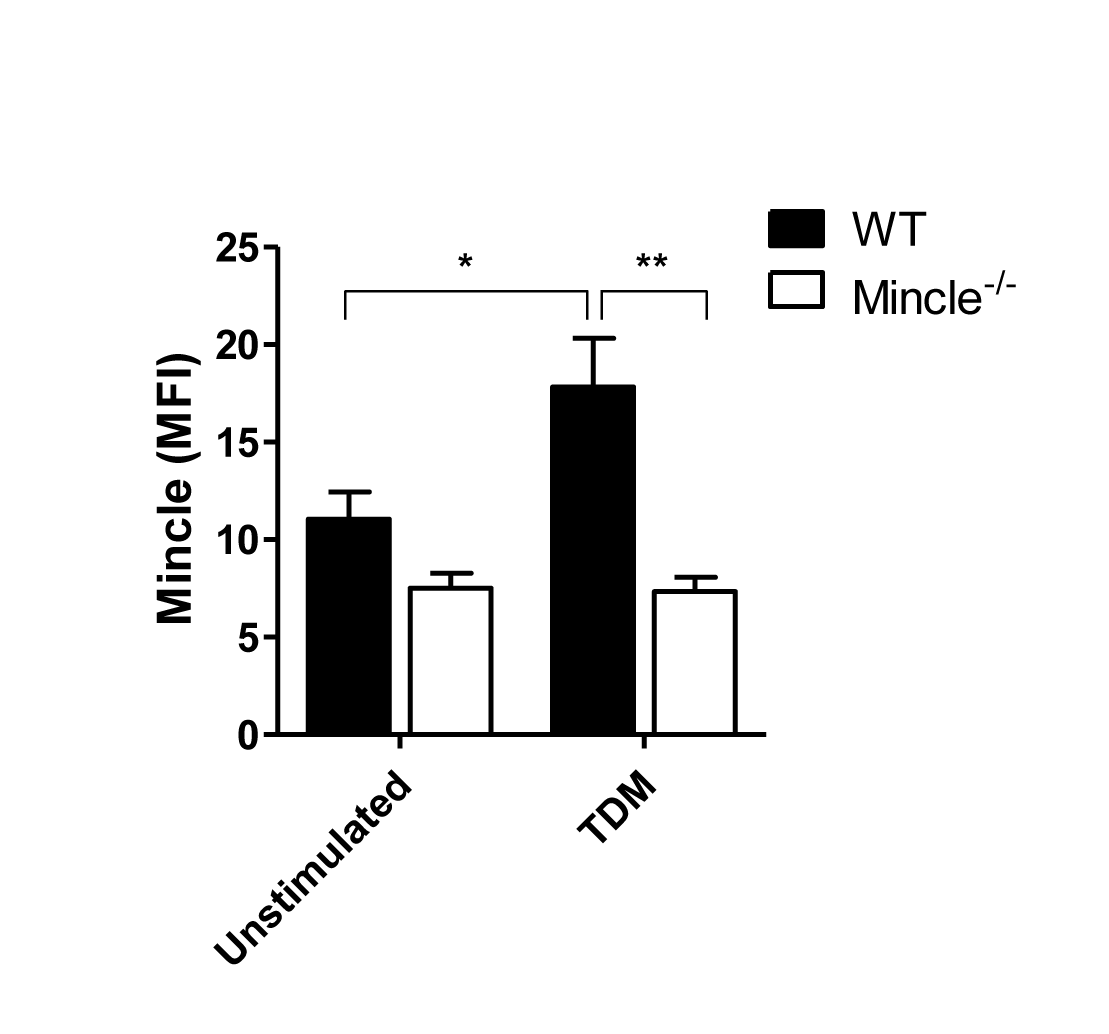

Supplement: Figure S1 — Surface expression of Mincle on neutrophils was elevated by TDM stimulation. Surface expression of Mincle was determined by flow cytometry. Neutrophils from wild-type (WT) and Mincle−/− mice were stimulated with 25 µg/ml trehalose dimycolate (TDM) for 18 h and analyzed by flow cytometry. Level of Mincle surface expression was quantified by mean fluorescence intensity (MFI). Statistical significance: *p<0.05 and **p<0.01. Data are expressed as means ± SEM from three independent experiments. (TIF) [file ppat.1002614.s001.tif]

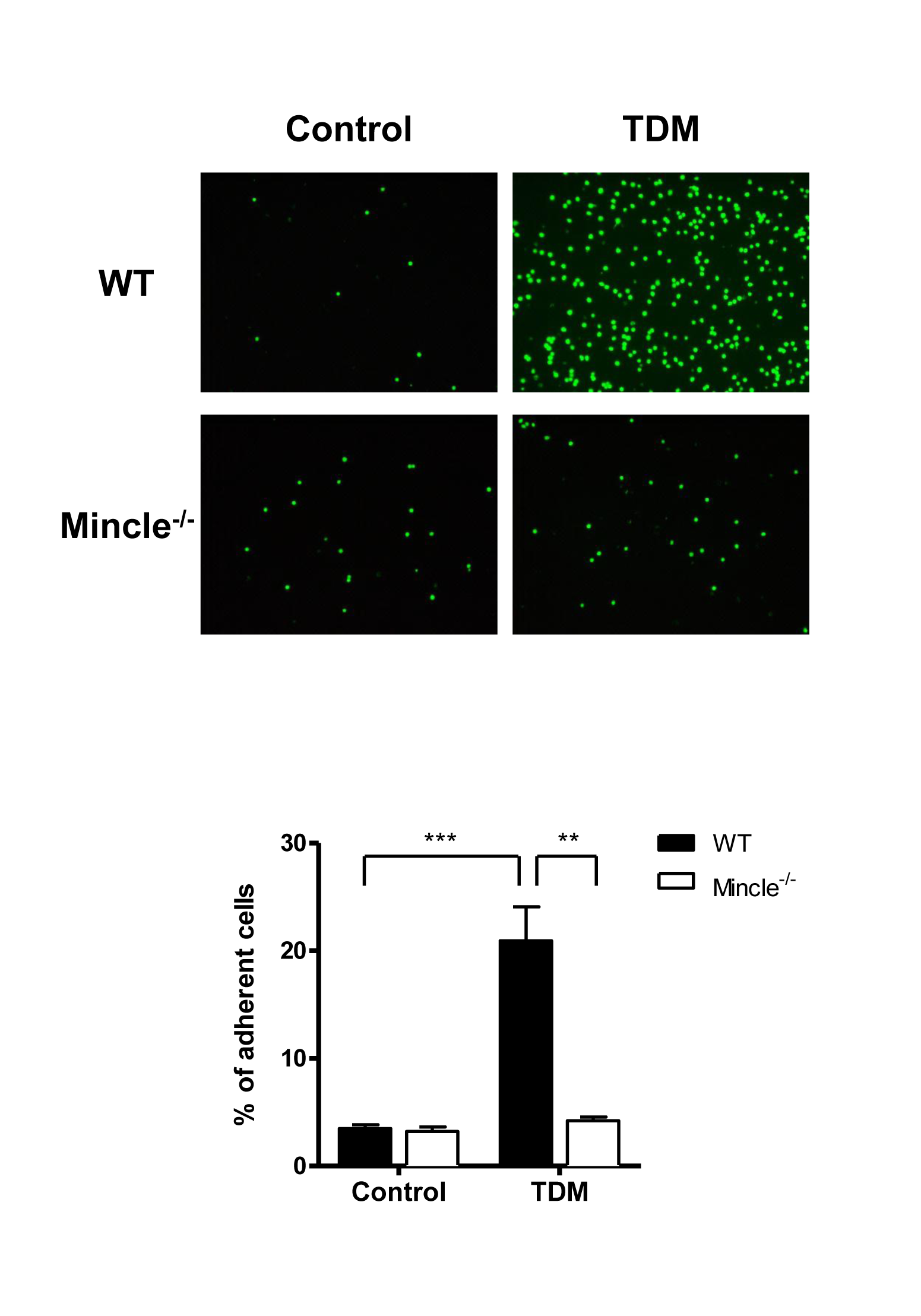

Supplement: Figure S2 — Neutrophil adhesion was increased by TDM stimulation. Suspended wild-type (WT) and Mincle−/− neutrophils were preincubated with calcein-acetoxymethyl ester at 37°C for 30 min. Then, the cells were incubated for 6 h on trehalose dimycolate (TDM)-coated plate. Nonadherent cells were removed, and then the adhered neutrophils were imaged by fluorescent microscopy. Original magnification was 200×. And fluorescence of each well was measured by using a fluorescence microplate reader (means ± SEM). Statistical significance: **p<0.01 and ***p<0.001. Data are expressed as means ± SEM from three independent experiments. (TIF) [file ppat.1002614.s002.tif]

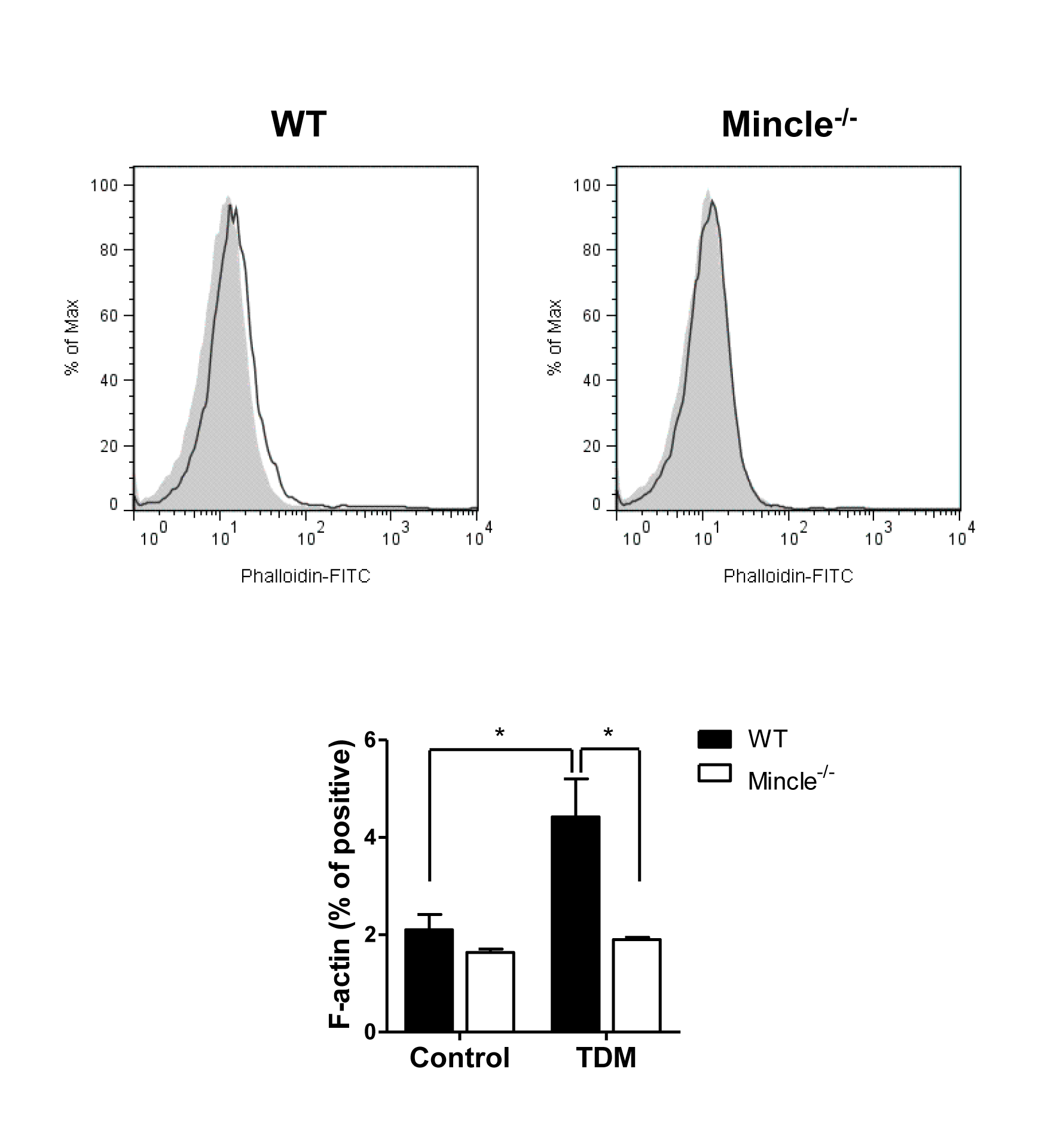

Supplement: Figure S3 — Actin polymerization was induced in response to TDM. Wild-type (WT) and Mincle−/− neutrophils were stimulated with trehalose dimycolate (TDM) for 18 h, and actin polymerization was measured by phalloidin staining and flow cytometry analysis. Results are given as mean ± SEM from three independent experiments. Statistical significance: *p<0.05. (TIF) [file ppat.1002614.s003.tif]

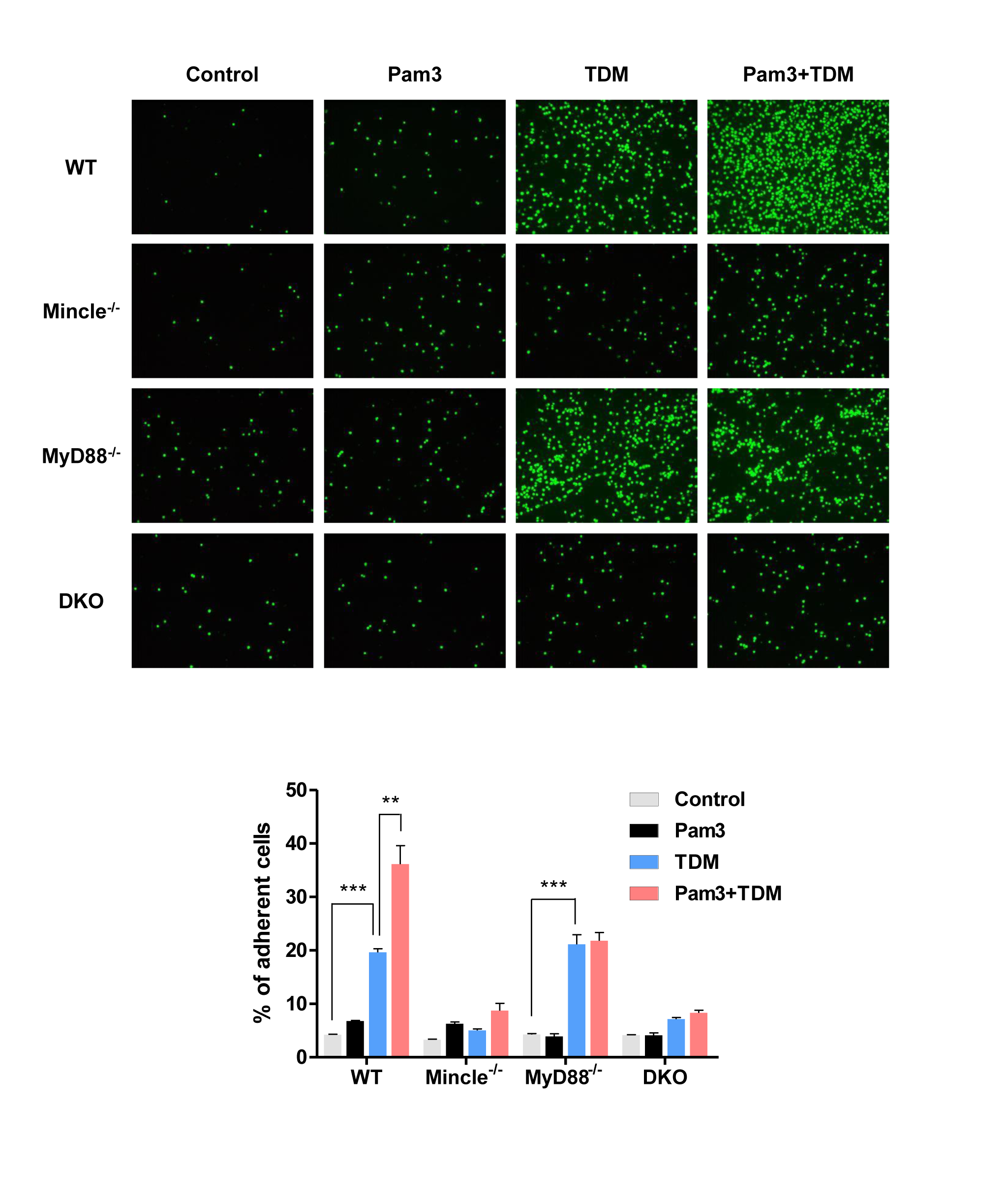

Supplement: Figure S4 — Cell adhesion was synergistically increased following co-stimulation with TDM/Pam3CSK4. Wild-type (WT), Mincle−/−, MyD88−/−, and Mincle−/−MyD88−/− (DKO) bone marrow neutrophils were preincubated with calcein-acetoxymethyl ester at 37°C for 30 min. Then, the cells were stimulated with Pam3CSK4 (10 ng/ml) and/or trehalose dimycolate (TDM, 25 µg/ml) for 6 h. The adhered neutrophils were imaged by fluorescent microscopy. Original magnification was 200×. And fluorescence of each well was measured by using a fluorescence microplate reader (means ± SEM). Statistical significance: **p<0.01 and ***p<0.001. Data are expressed as means ± SEM from three independent experiments. (TIF) [file ppat.1002614.s004.tif]
